# Subfamily-level comparative transcriptomics of key immune regulators in plants and suspension cells reveals novel rice blast resistance genes

**DOI:** 10.1093/pcp/pcag019

**Published:** 2026-02-10

**Authors:** Wanqing Wang, Fumi Fukada, Tomoyuki Furuta, Alfino Sebastian, Kiwamu Hyodo, Natsuko Ono, Hideki Nishimura, Pingyu Wang, Yoji Kawano

**Affiliations:** Institute of Plant Science and Resources, Okayama University, Chuo 2-20-1, Kurashiki, 710-0046 Okayama, Japan; Institute of Plant Science and Resources, Okayama University, Chuo 2-20-1, Kurashiki, 710-0046 Okayama, Japan; Institute of Plant Science and Resources, Okayama University, Chuo 2-20-1, Kurashiki, 710-0046 Okayama, Japan; Institute of Plant Science and Resources, Okayama University, Chuo 2-20-1, Kurashiki, 710-0046 Okayama, Japan; Institute of Plant Science and Resources, Okayama University, Chuo 2-20-1, Kurashiki, 710-0046 Okayama, Japan; Institute of Plant Science and Resources, Okayama University, Chuo 2-20-1, Kurashiki, 710-0046 Okayama, Japan; Institute of Plant Science and Resources, Okayama University, Chuo 2-20-1, Kurashiki, 710-0046 Okayama, Japan; Key Laboratory of Plant Hormones and Development Regulation of Chongqing, School of Life Sciences, Chongqing University, Chongqing 401331, China; Institute of Plant Science and Resources, Okayama University, Chuo 2-20-1, Kurashiki, 710-0046 Okayama, Japan

**Keywords:** DEGs, immunity, *Magnaporthe oryzae*, MAMP, rice, transcriptional landscape

## Abstract

Plants activate pattern-triggered immunity through key immune components, including pattern recognition receptors (PRRs), receptor-like cytoplasmic kinases (RLCKs), and transcription factors (TFs), to combat pathogens. However, a comprehensive transcriptional overview of these immune regulators at the subfamily level during biotic stress in rice is currently lacking. The aims of this study were to characterize the expression profiles of *Oryza sativa* (Os)PRRs, OsRLCKs, and OsTFs, and establish a robust pipeline for selecting novel candidate genes involved in plant immunity. We identified differentially expressed genes (DEGs) within these families using transcriptomic data from both rice plants infected with *Magnaporthe oryzae* infection and rice suspension cells subjected to chitin treatment. Our analysis revealed the transcriptional regulation of well-known immune-related subfamilies of OsPRRs, OsRLCKs, and OsTFs, such as receptor-like kinase-leucine-rich repeat XII (RLK-LRR-XII) and RLCK-VII, and identified several novel subfamilies with high proportions of DEGs that may contribute to pathogen perception and plant defense. We demonstrated that selecting candidates from overlapping DEGs between plant and suspension cell systems is an effective strategy for screening genes involved in rice immunity. Using this pipeline, novel immune regulators were identified, and their functions were confirmed. Two RLCKs, i.e. *OsRLCK298* and *OsBSR1*, act as positive regulators of immunity against rice blast fungus, whereas two TFs, i.e. *OsERF65* and *OsERF96.2*, act as negative regulators. This study provides a valuable transcriptomic resource and establishes a validated pipeline for gene discovery that could be applied to other stress responses and in other plant species.

## Introduction

Plant pathogens are responsible for global crop losses estimated at 20%–40%, translating to an annual economic impact of ~$200 billion (Savary et al. 2019). Consequently, enhancing crop immunity through genetic improvements is a cornerstone of global food security. The primary strategy involves identifying crucial disease-resistance genes for their subsequent integration into elite crop varieties. Forward genetics is a widely used approach that involves generating a mutagenized population and performing large-scale phenotypic screening to identify causal mutations. However, its efficacy is often hampered by the functional redundancy of homologous genes (Brutnell 2002). Recently, RNA-seq has become a powerful tool for generating genome-wide expression data (Wang et al. 2009). Although popular downstream analyses, such as Gene Ontology (GO) term enrichment, can predict gene functions, their utility is limited by the incompleteness of annotation databases. Furthermore, experimentally validating the function of numerous candidate genes identified through these methods remains a significant challenge (Khatri and Drăghici 2005, Rhee and Mutwil 2014). Therefore, a more refined and robust framework is needed to bridge the gap between high-throughput data and precise identification of validated plant resistance genes.

Rice blast, caused by the fungus *Magnaporthe oryzae* (*M. oryzae*) (anamorph *Pyricularia oryzae*), is one of the most destructive diseases of cultivated rice, causing annual production losses equivalent to the amount needed to feed 60 million people (Nalley et al. 2016). Plants use pattern recognition receptors (PRRs) located on the plasma membrane that perceive microbe-associated molecular patterns (MAMPs) to detect such pathogens. Recognition of MAMPs by PRRs activates pattern-triggered immunity (PTI) (Boller and Felix 2009), which is essential for curbing the spread of pathogens and improving plant survival. PRRs transduce pathogen signals to downstream receptor-like cytoplasmic kinases (RLCKs) (Macho and Zipfel 2014, Shiu and Bleecker 2001). The activation of RLCKs by PRRs triggers PTI responses such as defense-related gene expression through transcription factors (TFs), reactive oxygen species (ROS) production, and mitogen-activated protein kinase (MAPK) activation (Liang and Zhang 2022). These three key components, i.e. PRRs, RLCKs, and TFs, play important roles in plant immunity and function as signaling modules to prevent further pathogen invasion. Previous work supports their individual roles in stress responses. For instance, analysis of receptor-like kinase (RLK) expression in *Arabidopsis* revealed that 10 subfamilies are highly induced by several different biotic stress conditions (Lehti-Shiu et al. 2009). RLCKs exhibit significant differential expressions across various reproductive developmental stages and in response to cold, drought, and salt conditions in rice (Vij et al. 2008). Furthermore, expression analysis of the bHLH (basic helix–loop–helix) TF family in response to bacterial blight [*Xanthomonas oryzae* pv. *Oryzae* (*Xoo*)] led to the identification of two candidates of the negative regulators of bacterial blight resistance in rice (Zhang et al. 2023). Despite their importance, previous transcriptomic studies analyzing PRRs, RLCKs, and TFs have analyzed only one or two of the three gene families or focused on specific gene subsets within each gene family in different experimental conditions. Therefore, the aims of this study were to help decipher the comprehensive transcriptional landscape of PRR, RLCK, and TF candidates during the PTI response to better understand their regulation and identify potential targets for improving crop defense and securing food production. Moreover, integrating these transcriptional profiles with subfamily-level classifications offers a powerful framework for characterizing immune gene families and identifying high-priority candidates for functional validation.

PRRs include RLKs and receptor-like proteins (RLPs) (Lehti-Shiu et al. 2009). RLKs contain an ectodomain (ECD), a transmembrane domain, and a cytoplasmic kinase domain that can phosphorylate downstream RLCKs (Shiu and Bleecker 2001, Macho and Zipfel 2014). RLPs share a structure similar to that of RLKs, although they lack a kinase domain (Fritz-Laylin et al. 2005). PRRs can be classified into different subfamilies based on their ECD type. These ECDs allow the PRRs to perceive diverse signals (Tang et al. 2017). Although they are the cornerstones of plant immunity, PRRs have additional functions in plant growth, development, and reproduction (Ngou et al. 2024). MAMP treatment induces the expression of many PRR genes, and these transcriptional changes are often closely associated with immune function (Zipfel et al. 2004, Hou et al. 2014). Thus, the transcriptional profiles of PRRs can serve as functional indicators for candidate gene selection. For RLK subfamilies involved in plant immunity, members of the leucine-rich repeat (LRR) subfamily, especially those in the LRR-XII subfamily, are involved in MAMP perception. For example, flagellin, a protein subunit that forms the bacterial flagellum, is recognized by the FLAGELLIN SENSITIVE 2 (FLS2). Similarly, elongation factor-Tu (EF-Tu), an abundant and essential protein in bacterial translation, is perceived by its corresponding receptor, EFR (Boller and Felix 2009). Among the RLP subfamilies, LRR and LysM are among the few reported to be involved in plant immunity (Tang et al. 2017).

MAMP-induced oligomerization between PRRs is a common mechanism of MAMPs (Li et al. 2016). These receptor–kinase complexes are also involved in plant immunity. For instance, an LRR-RLP in *Arabidopsis*, named “RLP23,” is responsible for perceiving nlp20, a conserved 20-amino-acid fragment. RLP23 must form a signaling complex with two essential LRR-RLK coreceptors, i.e. SOBIR1 and BAK1, to initiate an immune response (Albert et al. 2015). XEG1, a protein produced by the pathogen *Phytophthora sojae*, is recognized by Response to XEG1 (RXEG1), LRR-RLP, and LRR-RLK coreceptors SOBIR1 and BAK1 in *Nicotiana benthamiana* (Sun et al. 2022b). Chitin is widely used as a model fungal MAMP. Although chitin perception has been extensively characterized in *Arabidopsis* and rice, the underlying mechanisms differ notably between these two species. Chitin perception in *Arabidopsis* is mediated exclusively by RLKs. Chitin induces heterodimerization of the receptor AtLYK5 and coreceptor AtCERK1 to initiate downstream signaling. Both are LysM-containing RLKs (Wan et al. 2012, Cao et al. 2014, Gong et al. 2020). In contrast, rice systems require both RLKs and RLPs. The rice RLK OsCERK1 cannot bind chitin and must form a complex with RLP, including chitin elicitor-binding protein (OsCEBiP), OsLYP4, or OsLYP6, to activate signaling (Shimizu et al. 2010, Liu et al. 2012a, Ao et al. 2014, Hayafune et al. 2014). All three OsRLPs belong to the LysM-containing glycosylphosphatidylinositol (GPI)-anchored protein subfamily. Despite the characterization of the foundational RLK–RLP complexes in rice, systematic characterization of these receptor pairs is required to fully harness their potential for crop protection. Identification of additional immune-related receptor pairs could provide new targets for engineering broad-spectrum disease resistance.

RLCKs play an essential role in the delivery of PTI to the cytoplasm. PRR–RLCK associations are key signaling modules in many biological pathways. Unlike PRRs, RLCKs lack an ECD; thus, they physically and functionally associate with RLKs to initiate downstream responses that regulate various biological functions. Phosphorylation of RLCKs by RLKs is essential for RLCK-mediated signaling (Liang and Zhou 2018, Hailemariam et al. 2024). RLKs and RLCKs are involved in plant immunity, growth, development, and reproduction (Liang and Zhou 2018). In total, 149 AtRLCKs and 379 OsRLCKs have been previously reported (Shiu et al. 2004, Vij et al. 2008). *Arabidopsis* and rice RLCKs have been divided into 17 subfamilies (Shiu et al. 2004, Liang and Zhou 2018). Members of the RLCK-VII subfamily are particularly well-known for their involvement in PTI, and their transcriptional regulation is often crucial for plant defense. For instance, reducing the expression of tomato RLCK-VII *TPK1b* via RNA interference compromises the resistance to the fungal pathogen *Botrytis cinerea* (AbuQamar et al. 2008). Similarly, a key member of *Arabidopsis* RLCK-VII, BOTRYTIS-INDUCED KINASE 1 (*BIK1*), was first identified through transcriptional analysis because its expression was highly induced in *Botrytis*-inoculated leaves (Veronese et al. 2006). *BIK1* directly interacts with RLKs, such as FLS2, EFR, and PEPR1, and is phosphorylated by RLKs to activate immunity (Hailemariam et al. 2024). *BIK1* also directly interacts with WRKY33, WRKY50, and WRKY57 to negatively regulate salicylic acid– and jasmonic acid–mediated immunity (Lal et al. 2018). OsRLCK185 and OsRLCK176 positively regulate chitin-induced immune responses in rice (Yamaguchi et al. 2013, Ao et al. 2014). OsRLCK57, OsRLCK107, and OsRLCK118, which are close homologs of OsRLCK176, positively regulate chitin-induced immunity (Li et al. 2017). Although RLCKs play a central role in chitin-triggered immune responses, specifying the exact contributions of most RLCK members remains challenging because of their large gene numbers and functional redundancy.

TFs are master regulators in the nucleus and are associated with various signaling pathways, such as plant development, stress management, and metabolic pathways (Chowdhary et al. 2023). In total, 58 TF families have been identified in higher plants (Jin et al. 2017). Six major TF families—AP2/ERF (APETALA2/ethylene-responsive factor), bHLH, MYB (myeloblastosis-related), NAC [no apical meristem (NAM), *Arabidopsis* transcription activation factor (ATAF1/2), and cup-shaped cotyledon (CUC2)], WRKY, and bZIP (basic leucine zipper)—are involved in plant defense (Ng et al. 2018). The gene numbers for each TF family in *Arabidopsis* and rice are AP2/ERF (*At*: 146, *Os*: 158), bHLH (*At*: 153, *Os*: 156), MYB (*At*: 205, *Os*: 191), NAC (*At*: 113, *Os*: 141), WRKY (*At*: 72, *Os*: 102), and bZIP (*At*: 74, *Os*: 96) (Ng et al. 2018). Looking specifically at the six TF families involved in rice immunity against *M. oryzae*, NAC and WRKY contain the most reported *OsTFs* (Park et al. 2025). For instance, the overexpression of *OsNAC6*, *OsNAC111*, and *OsWRKY53* enhances resistance (Chujo et al. 2007, Nakashima et al. 2007, Yokotani et al. 2014). Conversely, silencing of *OsNAC122* and *OsNAC131* or knockout of *OsWRKY55* compromises defense responses and increases susceptibility (Sun et al. 2013, Wang et al. 2023). Despite the large number of genes involved, few OsAP2/ERFs have been reported to participate in rice immunity. OsERF922 functions as a negative regulator of immunity against *M. oryzae* (Liu et al. 2012b). Conversely, *OsERF83* is a positive regulator because its overexpression suppresses lesion formation and increases expression of defense genes (Tezuka et al. 2019).

We integrated subfamily-level classification with comprehensive transcriptional profiling of the rice immune response to dissect the specific roles of the large PRR, RLCK, and TF gene families in plant defense. This integrated approach successfully identified subfamilies with enriched expression of defense-responsive genes, confirming the roles of known groups such as RLK-LRR-XII and RLCK-VII, and uncovered novel candidates, including LRR-VIII-1, LRR-XI-2, and RKF3. Moreover, we developed an effective pipeline using overlapping differentially expressed genes (DEGs) between plants and suspension cells to screen novel genes involved in rice blast resistance and confirmed that several high-priority candidate genes, including *OsRLCK298*, *OsBSR1*, *OsERF65*, and *OsERF96.2*, are genuine regulators of rice immunity. Taken together, our work provides a valuable transcriptomic resource for rice immunity and validates the use of overlapping DEGs as a robust method for gene discovery. This approach is broadly applicable to other stress responses and plant species.

## Results

### List for OsPRR candidates, OsRLCK, and OsTF subfamily/family transcripts, and an overview of their DEG distribution

**Figure 1:**
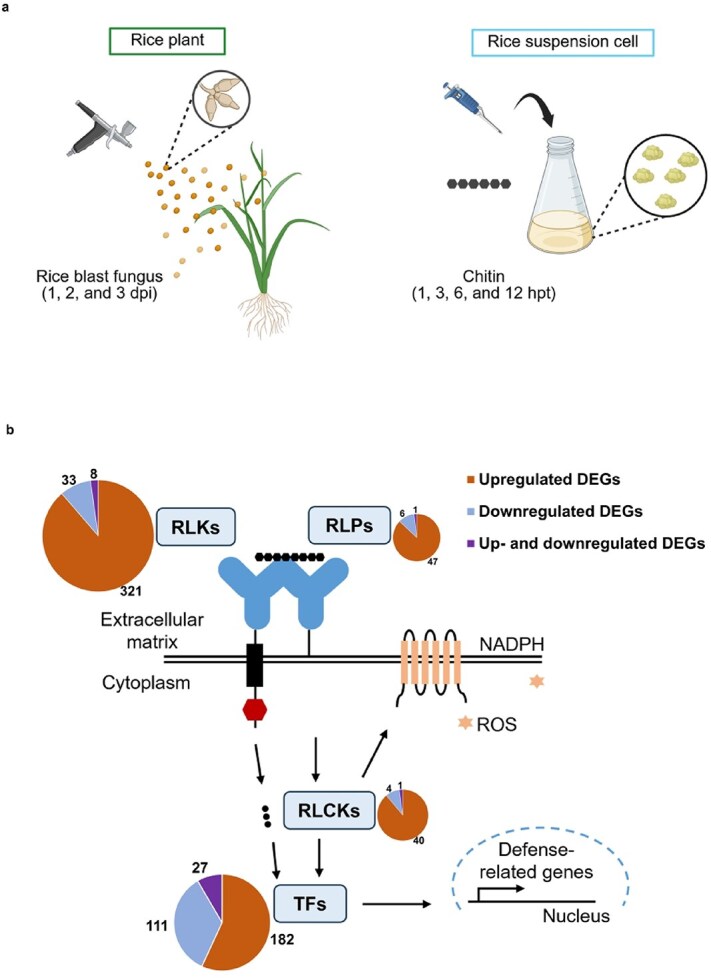
An overview of PRR, RLCK, and TF DEGs in the rice plant and suspension cell experiments. (a) Rice plants were infected with *M. oryzae* and collected at 1, 2, and 3 dpi. Rice suspension cells were treated with chitin and collected at 1, 3, 6, and 12 hpt. (b) OsRLK, OsRLP, OsRLCK, and OsTF DEGs were categorized into upregulated DEGs, downregulated DEGs, or up- and down-regulated DEGs. Upregulated DEGs and downregulated DEGs are defined as genes that are exclusively upregulated or downregulated, respectively, across all time points where they show significant differential expression. The category up- and down-regulated DEGs refers to genes that exhibit a dynamic expression pattern, being upregulated at certain time points and downregulated at others.

One objective of this study was to analyze the transcriptional responses of key signaling components, OsPRR candidates, OsRLCKs, and OsTFs, during immune responses. Here, we demonstrated an approach that combines transcriptional profiling with subfamily analysis to reliably identify candidate genes from key components that participate in the immune response to *M. oryzae*. We obtained the transcript lists of OsPRRs, OsRLCKs, and OsTFs (Supplementary Tables S1–S4), as described in the Materials and Methods. Within each key component, transcripts were grouped by subfamily/family and arranged according to transcript number in descending order (Table 1). In total, we identified 1153 OsRLKs, 175 OsRLPs, 210 OsRLCKs, and 2442 OsTFs. To study how pathogens and MAMPs affect gene expression, we analyzed two different experimental systems (Fig. 1a) (Wang et al. 2020a). First, we inoculated rice plant leaves with a virulent race of the rice blast fungus *M. oryzae* (007.0) and harvested them at 1, 2, and 3 days postinoculation (dpi). Second, we treated rice suspension cells with chitin and harvested them at 1, 3, 6, and 12 hours posttreatment (hpt). Principal component analysis (PCA) confirmed that the *M. oryzae* and chitin-treated samples clustered distinctly from each other and their respective mock controls (Supplementary Fig. S1). We performed bioinformatics analysis of the RNA-seq data to identify DEGs at each time point. DEGs were determined by comparing treated versus mock samples and were defined as genes with a false discovery rate (FDR or *P*_adj_) < 0.05 and an absolute log2FoldChange > 1. These DEGs were subsequently filtered to isolate the members of the OsPRR, OsRLCK, and OsTF families (Fig. 1b). A total of 362 OsRLK, 54 OsRLP, 45 OsRLCK, and 320 OsTF DEGs were identified. Most of the OsPRR and OsRLCK DEGs were upregulated in the rice plant and suspension cell experiments. In contrast, a higher proportion (35% or more) of the DEGs were downregulated in the OsTFs.

**Table 1:** Distribution of OsRLK, OsRLP, OsRLCK, and OsTF transcripts by subfamilies/families

RLK_subfamily	Transcript_num	RLP_subfamily	Transcript_num	RLCK_subfamily	Transcript_num	TF_family	Transcript_num
**DLSV**	187	**LRR**	98	**RLCK-VIIa-2**	48	**AP2/ERF**	188
**WAK**	131	**L-LECTIN**	25	**RLCK-IXb**	36	**bHLH**	181
**SD-2b**	115	**GPI_ANCHORED**	20	**RLCK-VIIa-1**	19	**NAC**	161
**LRR-XII-1**	114	**MALECTIN**	9	**RLCK-VIII**	18	**C2H2**	149
**L-LEC**	103	**WAK**	5	**RLCK-V**	17	**bZIP**	132
**LRR-XI-1**	61	**STRESS-ANTIFUNG**	5	**RLCK-VI**	13	**WRKY**	128
**LRR-III**	54	**G-LECTIN-B_G-LECTIN-P_G-LECTIN-S**	4	**RLCK-Os**	11	**MYB**	125
**LRK10L-2**	49	**LRR_MALECTIN**	2	**RLCK-IV**	9	**MYB-related**	97
**LRR-I-1**	44	**G-LECTIN-P_G-LECTIN-S**	2	**RLCK-XII-1**	8	**FAR1**	85
**CrRLK1L-1**	27	**G-LECTIN-B**	1	**RLCK-IXa**	6	**C3H**	83
**LRR-Xb-1**	25	**G-LECTIN-B_G-LECTIN-S**	1	**RLCK-XVI**	6	**GRAS**	69
**LRR-VIII-1**	23	**G-LECTIN-P**	1	**RLCK-XIII**	5	**B3**	65
**PERK-1**	20	**PR5K**	1	**RLCK-XV**	4	**HB-HD-ZIP**	59
**LRR-II**	20	**EGF**	1	**RLCK-X**	3	**MADS-MIKC**	58
**LRR-V**	19	**sum**	175	**RLCK-XI**	3	**GARP-G2-like**	57
**LRR-VI-2**	19			**RLCK-II**	2	**B3-ARF**	48
**CR4L**	17			**RLCK-VIIb**	2	**MADS-M-type**	40
**LysM**	15			**sum**	210	**LBD**	39
**Extensin**	13					**HSF**	38
**WAK_LRK10L-1**	12					**C2C2-Dof**	37
**others**	85					**Trihelix**	36
**sum**	1153					**C2C2-GATA**	32
						**OFP**	31
						**SBP**	29
						**TUB**	28
						**NF-YA**	25
						**TCP**	23
						**HB-other**	23
						**Tify**	23
						**PLATZ**	21
						**CPP**	20
						**HB-BELL**	20
						**others**	292
						**sum**	2442

### OsRLK and OsRLP subfamily distribution and expression of overlapping DEGs in the rice plant and suspension cell experiments

**Figure 2:**
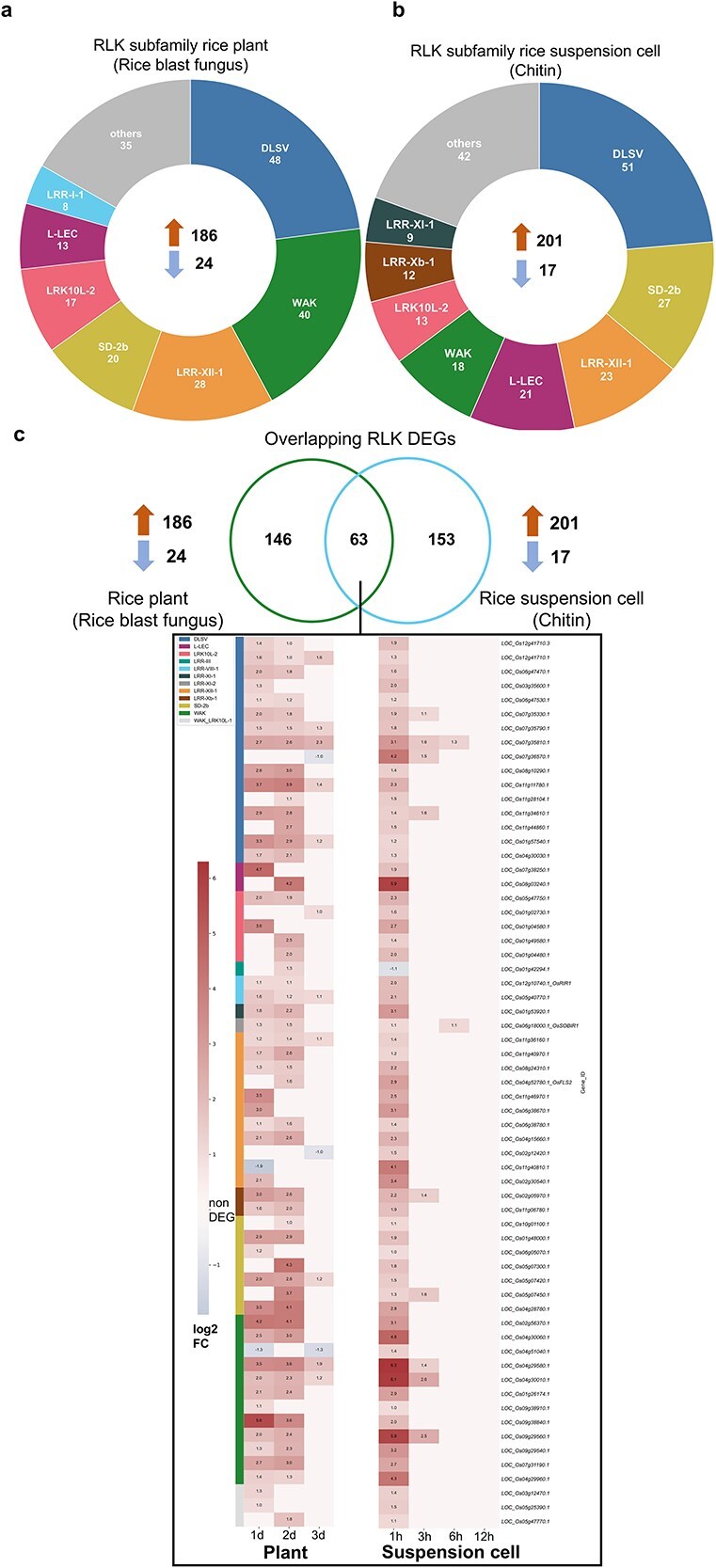
OsRLK DEG distribution by subfamily and gene expression profiles of overlapping DEGs in the rice plant and suspension cell experiments. OsRLK DEG subfamilies in the rice plant (a) and the suspension cell (b) experiments. (c) Overlapping OsRLK DEGs and heatmap showing gene expression in log2-fold change. Upward arrows indicate upregulated DEGs; downward arrows indicate downregulated DEGs.

We examined the DEGs grouped by subfamily in the rice plant and suspension cell experiments to obtain a closer look at specific *OsPRR* candidate subfamilies involved in pathogen and MAMP responses. Starting with RLK DEG subfamilies, 186 RLKs were upregulated, whereas only 24 were downregulated in the rice plant experiment (Fig. 2a and Supplementary Table S5). The top four DEG subfamilies were DLSV (DUF26, SD-1, LRR-VIII, and VWA), WAK, LRR-XII-1, and SD-2b. For RLK DEG subfamilies in the suspension cell experiment (Fig. 2b and Supplementary Table S6), >200 RLKs were upregulated and 17 were downregulated. The top four DEG subfamilies were DLSV, SD-2b, LRR-XII-1, and L-LEC.

OsRLK DEGs contain 16 subfamilies (Gao and Xue 2012). We summarized the percentage of OsRLK DEGs by subfamily in the rice plant and suspension cell experiments to study whether certain subfamilies are specifically associated with pathogen and MAMP treatments, and thus are more likely to be involved in PTI responses (Table 2). Seventeen subfamilies contained ≥30% DEGs. This value was selected because it encompasses all the RLK subfamilies previously implicated in biotic stress responses (Lehti-Shiu et al. 2009). Consequently, we established a threshold of 30% DEGs by subfamily/family as a reference for the analyses presented here and in the following tables. Yellow highlights indicate the subfamilies implicated in biotic stress, blue indicates a subfamily involved in both development and disease resistance, and green indicates a subfamily involved in development (Lehti-Shiu et al. 2009). LRR-XII-1, which is well-known for its role in MAMP perception, had a relatively high percentage of DEGs (35%), most of which were upregulated (32%). OsCERK1 and AtCERK1 are the key LysM RLKs involved in chitin perception in rice and *Arabidopsis*, respectively (Yang et al. 2022). Despite having only 15 members, the LysM subfamily exhibited a high percentage of DEGs (40%), all of which were upregulated. In summary, our results support the importance of the LRR-XII and LysM subfamilies from previous studies and suggest that previously reported subfamilies, such as LRR-Xb-1, LRK10L-2, WAK_LRK10L-1, LRR-I-2, DLSV, WAK, SD-2b, L-LEC, and LRR-I-1 (Lehti-Shiu et al. 2009), are likely involved in biotic stress. We observed that the RLK subfamilies, which are known to be involved in biotic stress, consistently showed that >30% of their members were differentially expressed, primarily through upregulation. The involvement of LRR-VIII-1, LRR-XI-2, and RKF3 subfamilies in this immune response has not been previously reported. Therefore, our observation that these groups exhibit the same expression trend as known immunity-related subfamilies suggests that they also contribute to defense against *M. oryzae*.

**Table 2:** Summary of OsRLK DEG subfamily distribution in the rice plant and suspension cell experiments

RLK	Transcript #	DEG #	Upregulated DEG% by subfamily	Downregulated DEG% by subfamily	DEG% by subfamily	Upregulated DEGs, plant (blast fungus)	Downregulated DEGs, plant (blast fungus)	Upregulated DEGs, suspension cell (chitin)	Downregulated DEGs, suspension cell (chitin)
**URK-3**	1	1	0%	100%	100%	0	0	0	1
**LRR-Xb-1**	25	14	52%	4%	56%	3	1	12	0
**LRK10L-2**	49	25	45%	6%	51%	15	2	12	1
**WAK_LRK10L-1**	12	6	50%	0%	50%	3	0	6	0
**LRR-I-2**	4	2	50%	0%	50%	0	0	2	0
**LRR-IV**	4	2	0%	50%	50%	0	0	0	2
**DLSV**	187	83	42%	4%	44%	42	6	51	1
**LysM**	15	6	40%	0%	40%	2	0	4	0
**PERK-2**	8	3	0%	38%	38%	0	0	0	3
**WAK**	131	46	34%	2%	35%	37	3	18	0
**LRR-XII-1**	114	40	32%	4%	35%	23	5	23	0
**LRR-VIII-1**	23	8	35%	0%	35%	5	0	5	0
**SD-2b**	115	40	33%	2%	35%	18	2	27	0
**LRR-XI-2**	3	1	33%	0%	33%	1	0	1	0
**RKF3**	3	1	33%	0%	33%	0	0	1	0
**L-LEC**	103	32	31%	0%	31%	13	0	21	0
**LRR-I-1**	44	13	27%	2%	30%	8	0	4	1
**CR4L**	17	5	29%	0%	29%	3	0	2	0
**CrRLK1L-1**	27	6	11%	15%	22%	1	3	2	1
**LRR-XI-1**	61	13	18%	3%	21%	5	0	7	2
**LRR-VI-2**	19	4	11%	16%	21%	1	2	1	1
**LRR-VI-1**	6	1	17%	0%	17%	1	0	0	0
**LRR-II**	20	3	10%	5%	15%	2	0	0	1
**Extensin**	13	1	8%	0%	8%	1	0	0	0
**LRR-III**	54	4	6%	4%	7%	2	0	1	2
**LRR-V**	19	1	0%	5%	5%	0	0	0	1
**PERK-1**	20	1	5%	0%	5%	0	0	1	0
**others**	56	0	0%	0%	0%	0	0	0	0
**sum**	1153	362	-	-	31%	186	24	201	17

RLK subfamilies were arranged by DEG% by subfamily (DEG # divided by transcript #) in a descending manner. Subfamilies with no DEGs identified were grouped into others. Highlighting indicates subfamilies previously associated with specific functions: Yellow for biotic stress in plants; Orange for the LRR-XII (MAMP perception) and LysM (chitin perception) subfamilies; Blue for the LRR-II subfamily (development and disease resistance); Green for the LRR-V subfamily (development). RLK subfamilies were divided into two sections based on 30% DEG% by subfamily.

Relying solely on DEG information makes it challenging to select candidate genes for further experiments. We propose that the overlapping DEGs in the rice plant and suspension cell experiments are critical genes involved in rice immunity against *M. oryzae*. The induction of these genes by both MAMP treatment and pathogen inoculation indicated their involvement in core immune signaling pathways. This overlapping criterion strengthens the claim that these are true DEGs by reducing the probability of their detection by chance. Thus, we focused on overlapping DEGs to aid in the selection process and further reduce the number of candidate genes. We identified 63 overlapping OsRLK DEGs (Fig. 2c), almost all of which were upregulated in the rice plant and suspension cell experiments. Most overlapping DEGs in the rice plant experiment were observed at 1 and 2 dpi. In contrast, the transcriptional response was concentrated at 1 hpt in the suspension cell experiment.

**Figure 3:**
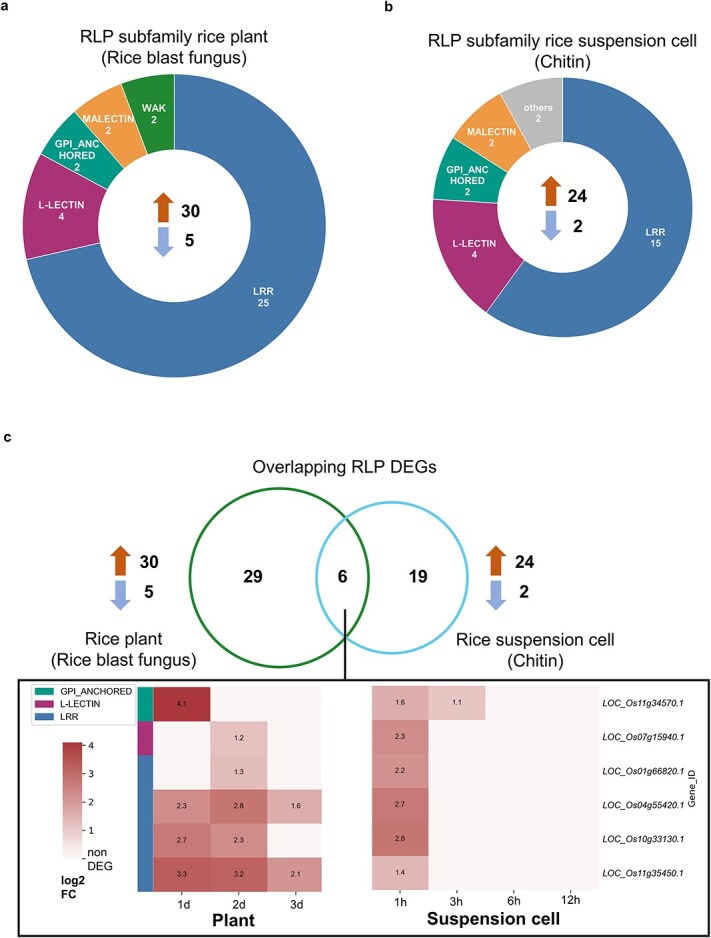
OsRLP DEG distribution by subfamily and gene expression profiles of overlapping DEGs in the rice plant and suspension cell experiments. OsRLP DEG subfamilies in the rice plant (a) and suspension cell (b) experiments. (c) Overlapping OsRLP DEGs and heatmap showing gene expression in log2-fold change. Upward arrows indicate upregulated DEGs; downward arrows indicate downregulated DEGs.

Next, we examined the composition of the OsRLP subfamily containing DEGs (Fig. 3a and b and Supplementary Tables S7 and S8). The majority of DEGs were upregulated in the rice plant and suspension cell experiments. We found that the LRR subfamily was the largest, followed by L-LECTIN, in both experiments. The four subfamilies with the highest percentages of DEGs were G-LECTIN-P_G-LECTIN-S, MALECTIN, WAK, and LRR (Table 3). Except for WAK, the remainder mainly contained upregulated DEGs. Six OsRLPs overlapped with DEGs in the rice plant and suspension cell experiments (Fig. 3c). All the overlapping DEGs were upregulated in both experiments. The DEGs were mainly distributed at 1 and 2 dpi in the rice plant experiment, whereas they were mainly distributed at 1 hpt in the suspension cell experiment.

**Table 3:** Summary of OsRLP DEG subfamily distribution in the rice plant experiment and the suspension cell experiments

RLP	Transcript #	DEG #	Upregulated DEG% by subfamily	Downregulated DEG% by subfamily	DEG% by subfamily	Upregulated DEGs, plant (blast fungus)	Downregulated DEGs, plant (blast fungus)	Upregulated DEGs, suspension cell (chitin)	Downregulated DEGs, suspension cell (chitin)
**G-LECTIN-P_G-LECTIN-S**	2	1	50%	0%	50%	0	0	1	0
**MALECTIN**	9	4	44%	0%	44%	2	0	2	0
**WAK**	5	2	20%	20%	40%	1	0	0	0
**LRR**	98	36	34%	3%	37%	22	3	15	0
**L-LECTIN**	25	7	28%	4%	28%	4	0	4	1
**G-LECTIN-B_G-LECTIN-P_G-LECTIN-S**	4	1	25%	0%	25%	0	0	1	0
**GPI_ANCHORED**	20	3	5%	10%	15%	1	1	1	1
**others**	12	0	0%	0%	0%	0	0	0	0
**sum**	175	54	-	-	31%	30	4	24	2

RLP subfamilies were arranged by DEG% by subfamily (DEG # divided by transcript #) in a descending order. Subfamilies with no DEGs identified were grouped into others. Yellow highlighting indicates subfamilies to be involved in biotic stress in plants. RLP subfamilies were divided into two sections based on 30% DEG% by subfamily.

### OsRLCK subfamily distribution in the rice plant and suspension cell experiments

**Figure 4:**
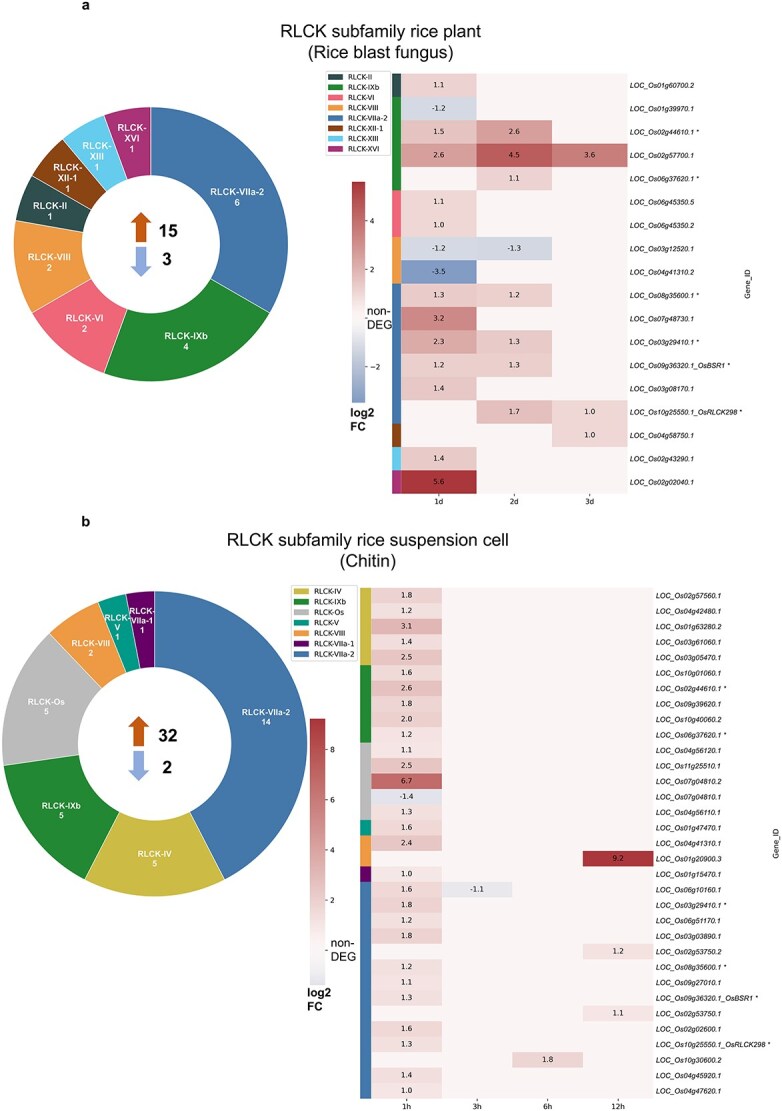
OsRLCK DEG distribution by subfamily and gene expression profiles of DEGs in the rice plant and suspension cell experiments. OsRLCK DEG subfamilies and heatmap showing gene expression in log2-fold change in the rice plant experiment (a) and the suspension cell (b) experiment. Asterisks (*) denote overlapping DEGs and candidate gene names. Upward arrows indicate upregulated DEGs; downward arrows indicate downregulated DEGs.

We grouped OsRLCK DEGs (Supplementary Tables S9 and S10) by subfamily and plotted their expression patterns for both the rice plant (Fig. 4a) and the suspension cell (Fig. 4b) experiments to examine whether certain subfamilies were preferentially induced by the pathogen and MAMP treatments. In the rice plant experiment, RLCK-VIIa-2 and RLCK-IXb were the top DEG subfamilies. Most OsRLCKs were upregulated at 1 and 2 dpi. In the suspension cell experiment, RLCK-VIIa-2 was the top DEG subfamily, followed by the RLCK-IV, -IXb, and -Os subfamilies. Six OsRLCKs overlapped with DEGs in the rice plant and suspension cell experiments (Supplementary Fig. S2a). Their expression levels in the rice plant and suspension cell experiments are shown in Fig. 4a and b (indicated by asterisks). All six overlapping DEGs were upregulated in both experiments. OsRLCK-VII members are involved in PTI responses (Rao et al. 2018, Jalilian et al. 2023). In addition to RLCK-VIIa-2, in which 33% of the members were DEGs, we observed that the RLCK-IV, -II, and -Os subfamilies had high proportions of DEGs, most of which were upregulated (Table 4). These results support the importance of RLCK-VII in *M. oryzae–*triggered immune responses and identify the RLCK-IV, -II, and -Os subfamilies as potential candidates for further investigation.

**Table 4:** Summary of OsRLCK DEG subfamily distribution in the rice plant and suspension cell experiments

RLCK	Transcript #	DEG #	Upregulated DEG% by subfamily	Downregulated DEG% by subfamily	DEG% by subfamily	Upregulated DEGs, plant (blast fungus)	Downregulated DEGs, plant (blast fungus)	Upregulated DEGs, suspension cell (chitin)	Downregulated DEGs, suspension cell (chitin)
**RLCK-IV**	9	5	56%	0%	56%	0	0	5	0
**RLCK-II**	2	1	50%	0%	50%	1	0	0	0
**RLCK-Os**	11	5	36%	9%	45%	0	0	4	1
**RLCK-VIIa-2**	48	16	33%	2%	33%	6	0	14	1
**RLCK-VIII**	18	4	11%	11%	22%	0	2	2	0
**RLCK-XIII**	5	1	20%	0%	20%	1	0	0	0
**RLCK-IXb**	36	7	17%	3%	19%	3	1	5	0
**RLCK-XVI**	6	1	17%	0%	17%	1	0	0	0
**RLCK-VI**	13	2	15%	0%	15%	2	0	0	0
**RLCK-XII-1**	8	1	13%	0%	13%	1	0	0	0
**RLCK-V**	17	1	6%	0%	6%	0	0	1	0
**RLCK-VIIa-1**	19	1	5%	0%	5%	0	0	1	0
**others**	18	0	0%	0%	0%	0	0	0	0
**sum**	210	45	-	-	21%	15	3	32	2

RLCK subfamilies were arranged by DEG% by subfamily (DEG # divided by transcript #) in a descending order. Subfamilies with no DEGs identified were grouped into others. Yellow highlighting indicates subfamilies to be involved in biotic stress in plants. RLCK subfamilies were divided into two sections based on 30% DEG% by subfamily.

### OsTF subfamily distribution and expression of overlapping DEGs in the rice plant and suspension cell experiments

**Figure 5:**
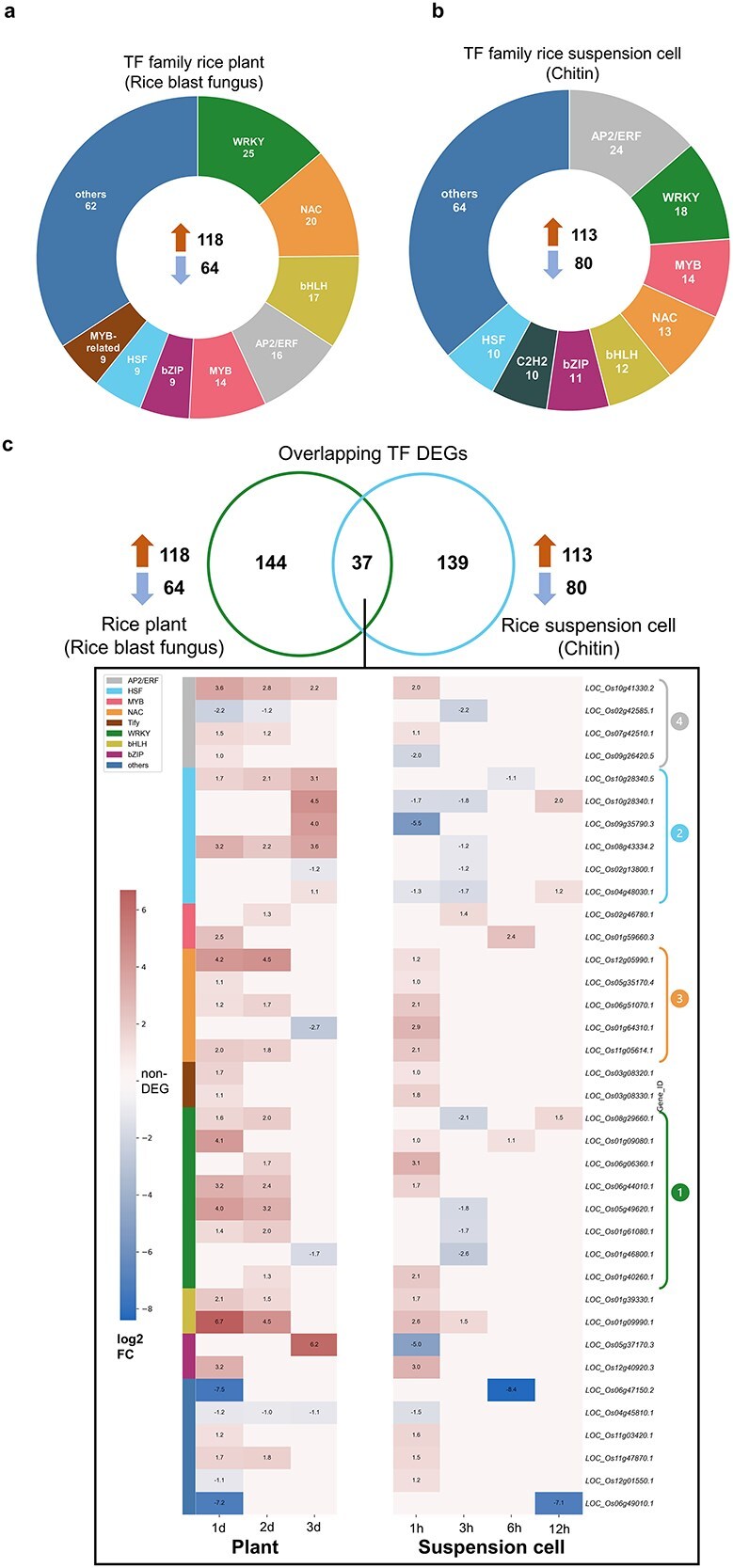
OsTF DEG distribution by family and gene expression profiles of overlapping DEGs in the rice plant experiment and suspension cell experiment. OsTF DEG families in the rice plant experiment (a) and the suspension cell (b) experiment. (c) Overlapping OsTF DEGs and heatmap showing gene expression in log2-fold change. 1. WRKY; 2. HSF; 3. NAC; and 4. AP2/ERF. Upward arrows indicate upregulated DEGs; downward arrows indicate downregulated DEGs.

TFs can aid the PTI response by regulating defense genes. We grouped OsTF DEGs (Supplementary Tables S11 and S12) into families in the rice plant (Fig. 5a) and suspension cell (Fig. 5b) experiments to identify TF families responsive to pathogen and MAMP treatments and to locate a candidate OsTF family for selecting candidate genes involved in immunity against *M. oryzae*. WRKY, NAC, bHLH, and AP2/ERF were the top four OsTF DEG families in the rice plant experiment. AP2/ERF, WRKY, MYB, and NAC were the top four OsTF DEG families identified in the suspension cell experiment. In addition to the TF families reported to be involved in biotic stress (Table 5; highlighted in yellow), we identified many other TF families that had a high percentage of DEGs. The proportions of upregulated and downregulated DEGs differed among different TF families.

**Table 5:** Summary of OsTF DEG family distribution in the rice plant and suspension cell experiments

TF	Transcript #	DEG #	Upregulated DEG% by family	Downregulated DEG% by family	DEG% by family	Upregulated DEGs plant (blast fungus)	Downregulated DEGs, plant (blast fungus)	Upregulated DEGs, suspension cell (chitin)	Downregulated DEGs, suspension cell (chitin)
**HSF**	38	13	26%	26%	34%	8	1	4	10
**E2F-DP**	10	3	10%	20%	30%	1	0	0	2
**SBP**	29	8	17%	10%	28%	3	2	2	2
**WRKY**	128	35	24%	5%	27%	23	2	13	6
**C2C2-LSD**	12	3	0%	25%	25%	0	3	0	0
**zf-HD**	14	3	14%	7%	21%	1	0	2	1
**MYB**	125	26	16%	5%	21%	13	1	9	5
**GARP-G2-like**	57	11	11%	11%	19%	3	4	3	2
**AP2/ERF**	188	36	14%	7%	19%	12	4	17	10
**EIL**	11	2	9%	18%	18%	1	1	0	1
**LBD**	39	7	10%	10%	18%	2	3	2	1
**TUB**	28	5	18%	0%	18%	1	0	4	0
**NAC**	161	28	14%	4%	17%	14	6	12	1
**Tify**	23	4	13%	4%	17%	3	1	2	0
**DBP**	6	1	17%	0%	17%	0	0	1	0
**OFP**	31	5	10%	6%	16%	2	0	1	2
**bHLH**	181	27	8%	8%	15%	7	10	9	4
**bZIP**	132	18	8%	7%	14%	6	3	6	6
**MYB-related**	97	13	7%	7%	13%	6	3	1	4
**C2C2-GATA**	32	4	3%	9%	13%	0	1	1	2
**MADS-MIKC**	58	7	2%	10%	12%	0	5	1	1
**C3H**	83	10	7%	6%	12%	3	1	3	4
**HB-HD-ZIP**	59	7	7%	5%	12%	0	3	4	1
**GRAS**	69	8	9%	3%	12%	2	1	5	1
**GRF**	19	2	5%	5%	11%	0	1	1	0
**DDT**	10	1	10%	10%	10%	0	0	1	1
**HB-BELL**	20	2	0%	10%	10%	0	2	0	0
**DBB**	11	1	9%	0%	9%	1	0	0	0
**Alfin-like**	11	1	0%	9%	9%	0	0	0	1
**C2H2**	149	13	6%	5%	9%	2	1	7	6
**TCP**	23	2	0%	9%	9%	0	0	0	2
**NF-YA**	25	2	8%	0%	8%	2	0	0	0
**RWP-RK**	14	1	0%	7%	7%	0	0	0	1
**HB-KNOX**	15	1	0%	7%	7%	0	0	0	1
**B3**	65	4	5%	2%	6%	1	1	2	0
**CPP**	20	1	0%	5%	5%	0	0	0	1
**PLATZ**	21	1	5%	0%	5%	1	0	0	0
**B3-ARF**	48	2	0%	4%	4%	0	2	0	1
**Trihelix**	36	1	0%	3%	3%	0	1	0	0
**MADS-M-type**	40	1	0%	3%	3%	0	1	0	0
**others**	304	0	0%	0%	0%	0	0	0	0
**sum**	2442	320	-	-	13%	118	64	113	80

TF families were arranged by DEG% by family (DEG # divided by transcript #) in a descending order. Families with no DEGs identified were grouped into others. Yellow highlighting indicates families to be involved in biotic stress in plants. TF families were divided into two sections based on 30% DEG% by family.

A total of 37 OsTFs were identified as overlapping DEGs in both experiments. We focused on the four families with the highest number of DEGs, i.e. WRKY, heat shock factor (HSF), NAC, and AP2/ERF, to select a candidate OsTF family for functional analysis (Fig. 5c). In the rice plant experiment, DEGs from the WRKY and NAC families were predominantly upregulated at 1 and 2 dpi. In the suspension cell experiment, all the NAC DEGs were upregulated at 1 hpt. In contrast, only half of the WRKY DEGs were upregulated at 1 hpt, whereas the remainder were downregulated at 3 hpt. The identification of numerous DEGs from the WRKY and NAC families was expected, given their previously reported involvement in *M. oryzae*–induced PTI responses. Notably, the HSF family also contained many DEGs; however, all of these were downregulated in the suspension cell experiment. HSF proteins regulate gene expression in response to various abiotic stresses, such as heat stress (Tao et al. 2024), which may explain this distinct expression pattern. Therefore, the HSF family was excluded from further analysis. The expression pattern of the AP2/ERF family was intermediate, with the majority of its DEGs upregulated at 1 and 2 dpi in plants and at 1 hpt in suspension cells. Compared to the well-studied WRKY and NAC families, the role of AP2/ERF in immunity against *M. oryzae* is less well characterized. Therefore, the AP2/ERF family was selected for further analysis.

**Figure 6:**
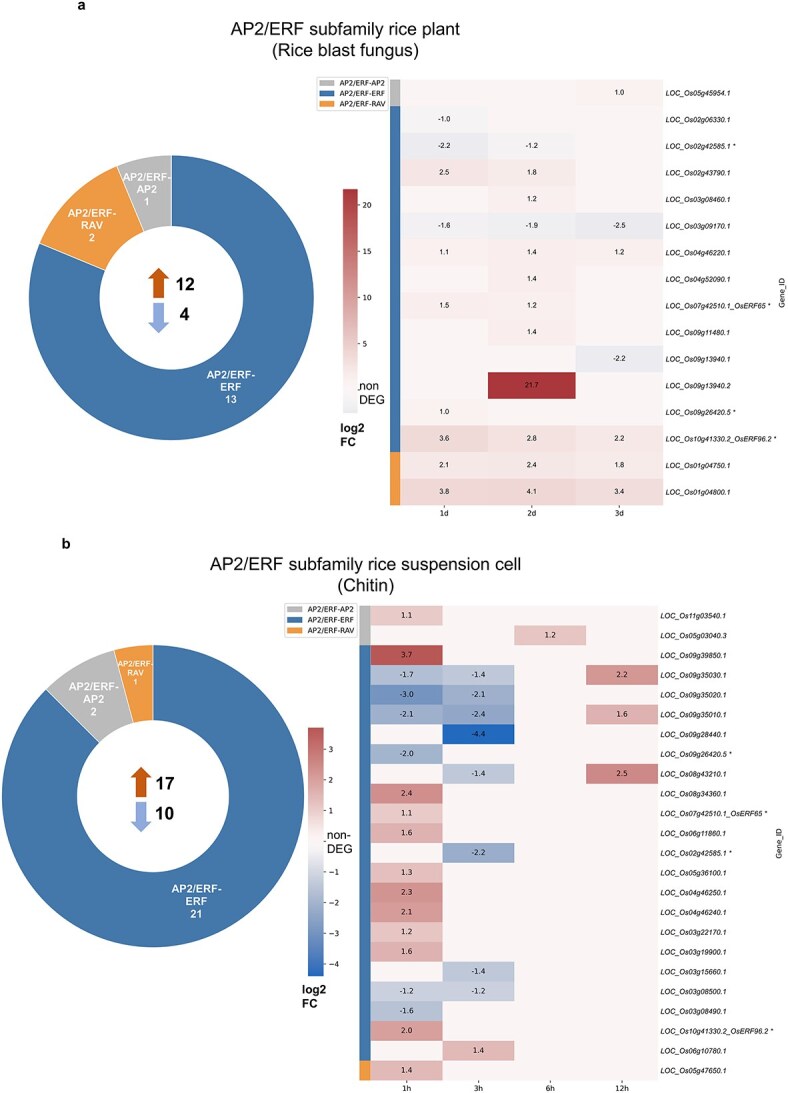
AP2/ERF DEG distribution by subfamily and gene expression profiles of DEGs in the rice plant experiment and suspension cell experiment. AP2/ERF DEG subfamilies and heatmap showing gene expression in log2-fold change in the rice plant (a) and the suspension cell (b) experiments. Asterisks (*) denote overlapping DEGs and candidate gene names. Upward arrows indicate upregulated DEGs; downward arrows indicate downregulated DEGs.

The AP2/ERF family in rice contains 188 transcripts, with ERF being the largest subfamily (165 members), followed by AP2 (19 members) and RAV (4 members) (Table 6). Based on the domain structure, the AP2/ERF family in *Arabidopsis* is classified into four subfamilies: AP2 (two AP2 domains), ERF (one AP2 domain), RAV (one AP2 domain and one B3 domain), and soloist (others) (Nakano et al. 2006). Following this classification, we analyzed the DEG subfamily distribution and gene expression profiles in the rice plant (Fig. 6a) and in the suspension cell (Fig. 6b) experiments. As the largest subfamily, the ERF contained the highest number of DEGs in both experiments. In the rice plant experiment, the DEGs were predominantly upregulated at 1 and 2 dpi. Upregulated DEGs in the suspension cell experiment were most abundant at 1 hpt, whereas downregulated DEGs were most abundant at 3 hpt. We identified four overlapping AP2/ERF DEGs between the two experiments (Supplementary Fig. S2b). The expression patterns of the overlapping genes are shown in Fig. 6a and b (indicated by asterisks).

**Table 6:** Summary of AP2/ERF subfamily distribution in the rice plant and suspension cell experiments

AP2/ERF	Transcript #	DEG #	Upregulated DEG% by subfamily	Downregulated DEG% by subfamily	DEG% by subfamily	Upregulated DEGs plant (blast fungus)	Downregulated DEGs, plant (blast fungus)	Upregulated DEGs, suspension cell (chitin)	Downregulated DEGs, suspension cell (chitin)
**RAV**	4	3	75%	0%	75%	2	0	1	0
**ERF**	165	30	13%	8%	18%	9	4	14	10
**AP2**	19	3	16%	0%	16%	1	0	2	0
**sum**	188	36	-	-	19%	12	4	17	10

AP2/ERF subfamilies were arranged by DEG% by subfamily (DEG # divided by transcript #) in a descending order. AP2/ERF subfamilies were divided into two sections based on 30% DEG% by subfamily.

In summary, we identified OsPRR, OsRLCK, and OsTF subfamilies/families that likely play important roles in pathogen recognition and defense responses. Furthermore, we generated a transcriptional landscape of the overlapping DEGs with subfamily information, which provided a strong foundation for candidate gene selection.

### Candidate gene selection for OsRLCKs and OsTFs involved in PTI

**Figure 7:**
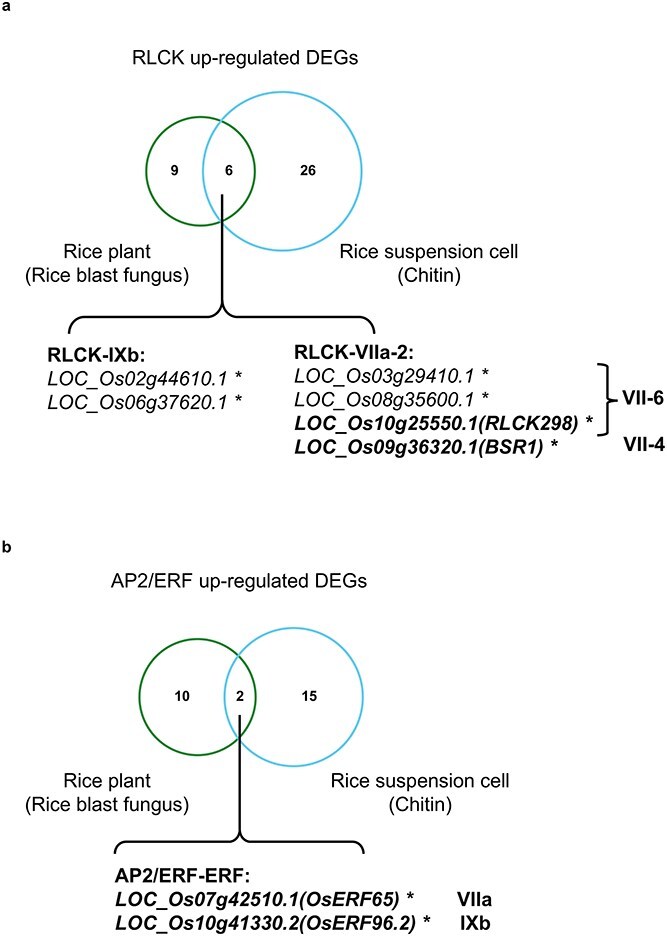
Candidate gene selection for OsRLCKs and AP2/ERF OsTFs*.* (a) Overlapping upregulated OsRLCKs consist of the RLCK-IXb and RLCK-VIIa-2 subfamilies, which were further categorized into subgroup 4 (VII-4) and subgroup 6 (VII-6). (b) Two OsERFs genes were identified as upregulated DEGs in both experiments.

Relying solely on differential gene expression profiles presents a challenge for effective candidate gene selection. Consequently, we prioritized the analysis of DEGs shared between the rice plant and suspension cell experiments to generate a more robust list of candidates. Analysis of overlapping DEGs provided a direct path for selecting candidate OsRLCK and OsAP2/ERF genes. Our selection strategy prioritized genes that were upregulated in both rice plant and suspension cell experiments. Six OsRLCK genes were upregulated in both assays (Fig. 7a). Based on a previous report that RLCK-VII-4 members are involved in chitin-triggered immunity, whereas RLCK-VII-6 members negatively regulate ROS signaling (Rao et al. 2018), we selected one candidate from each of these functionally relevant subgroups: *OsBSR1* (RLCK-VII-4) and *OsRLCK298* (RLCK-VII-6). Similarly, within the AP2/ERF TF family, two genes were consistently upregulated in both experiments. Therefore, *OsERF65* and *OsERF96.2* were selected as candidate genes for further analyses (Fig. 7b). *OsERF65* (VIIa) and *OsERF96.2* (IXb) were classified into different phylogenetic groups (Nakano et al. 2006).

### *OsRLCK298* and *OsBSR1* positively regulate rice immunity

**Figure 8:**
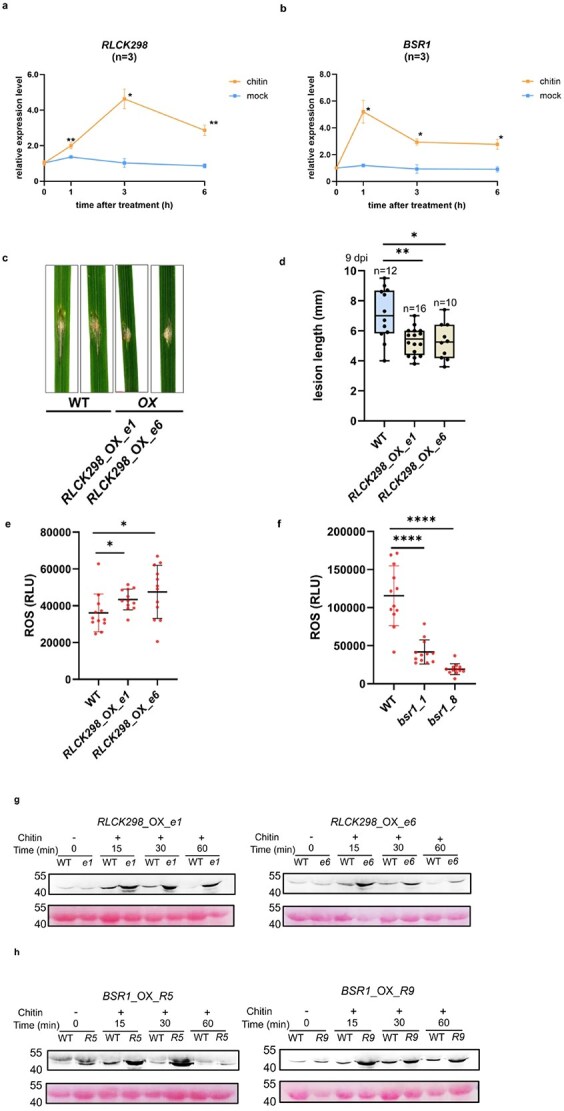
OsRLCK genes positively regulate rice immunity against *M. oryzae*. Relative expression of *OsRLCK298* (a) and *OsBSR1* (b) induced in suspension cells treated with chitin. Expression levels were quantified by quantitative reverse transcription PCR (qRT-PCR). Bars represent mean ± SD (*n* = 3 biological replicates; two-tailed *t*-test; ^*^P < 0.05; ^**^P < 0.01). The representative disease symptoms (c) and disease lesion length (d) on the inoculated leaves of the WT and *OsRLCK298_*OX transgenic lines after *M. oryzae* punch infection. Bars represent mean ± SD (*n* ≥ 10 biological replicates; unpaired *t*-test; ^*^P < 0.05; ^**^P < 0.01). Peak ROS induction of chitin minus mock in WT and *OsRLCK298_*OX (e) and *bsr1* (f) transgenic plants. Leaf discs were treated with 1 μg/mL chitin or mock (ddH_2_O). Bars represent mean ± SD (*n* = 12 biological replicates; unpaired *t*-test; ^*^  *P* < 0.05; ^****^P < 0.0001). Chitin-induced MAPK phosphorylation in leaf discs of the WT and *OsRLCK298_*OX (g) or *OsBSR1_*OX (h) lines.

Our initial transcriptomic analysis revealed that *OsRLCK298* and *OsBSR1* were significantly upregulated by *M. oryzae* and chitin, suggesting their potential involvement in rice immunity. We first confirmed that chitin treatment induces the expression of both genes to test this hypothesis (Fig. 8a and b). We performed punch infection assays using a virulent race (Ao92-06-02) of *M. oryzae* to assess their roles in pathogen defense (Fig. 8c and d). Attempts to generate *OsRLCK298* knockout (KO) lines were unsuccessful, likely due to lethality; therefore, we created overexpressing (OX) lines for this study (Supplementary Fig. S3a). The *OsRLCK298*_OX lines exhibited significantly shorter lesion lengths than the wild-type (WT) lines, indicating enhanced resistance. We measured the ROS burst to further investigate their function in PTI. Consistent with its positive regulatory role, peak ROS levels in the *OsRLCK298*_OX lines were modestly but significantly higher than those in the WT (Fig. 8e, *P* < 0.05). Correspondingly, and in agreement with previous studies showing *OsBSR1* is a positive regulator of blast resistance (Sugano et al. 2018), the ROS burst was significantly reduced in *bsr1* lines (Kanda et al. 2017) (Fig. 8f). These results demonstrated that both *OsRLCK298* and *OsBSR1* positively regulated MAMP-induced ROS production and resistance to *M. oryzae*. RLCKs function as central signaling hubs downstream of PRRs and are known to link pathogen perception to MAPK signaling cascades (Yamaguchi et al. 2013). To provide a direct mechanistic link for our positive-regulating RLCK candidates, we performed MAPK activation assays using rice plants (Fig. 8g and h). We found that while the overexpression of *OsRLCK298* or *OsBSR1* alone triggered minimal MAPK activation, it significantly enhanced chitin-induced phosphorylation of MAP kinases. These findings position the two RLCKs as key signaling components that amplify the chitin-activated immune cascade through the MAPK signaling.

### *OsERF65* and *OsERF96.2* negatively regulate rice immunity

We first confirmed that the expression of both *OsERF65* and *OsERF96.2* was induced by chitin treatment (Fig. 9a and b) to investigate the functions of the candidate OsERF genes. We generated KO lines for both, *erf65* and *erf96.2* (Supplementary Fig. S3b), and assessed their resistance to *M. oryzae* using a punch infection assay. Compared with the WT, lesion lengths were significantly reduced in both the *erf65* (Fig. 9c and d) and *erf96.2* lines (Fig. 9e and f), indicating that the loss of function of these genes enhances disease resistance. We quantified the ROS levels to determine whether this resistance was related to the initial MAMP-triggered ROS burst. However, no significant differences were observed between the WT and KO lines for either gene (Fig. 9g and h). Because these genes encode transcription factors, we examined their impact on defense-related gene expression following spray infection. The expression of *PR5* was significantly higher in the *erf65* lines than in the WT plants (Fig. 9i). The induction of *PR-1a*, *PR1b*, and *PR10* in *erf65-4* was significantly higher than that in the WT. Those in *erf65_1* showed a consistent trend of higher expression, but did not reach statistical significance. Similarly, defense gene induction was consistently higher in the *erf96.2* lines than in the WT, with at least one mutant line showing a statistically significant increase for each tested PR gene (Fig. 9j). To further investigate the immune mechanisms mediated by the candidate OsERF genes, we performed MAPK activation assays on the *erf65* and *erf96.2* KO plants. We observed that chitin-induced MAPK activation levels in the *erf65* and *erf96.2* KO plants were higher than those in the WT (Fig. 9k and l), suggesting that ERF65 and ERF96.2 function as negative regulators of chitin-triggered MAPK signaling.

**Figure 9:**
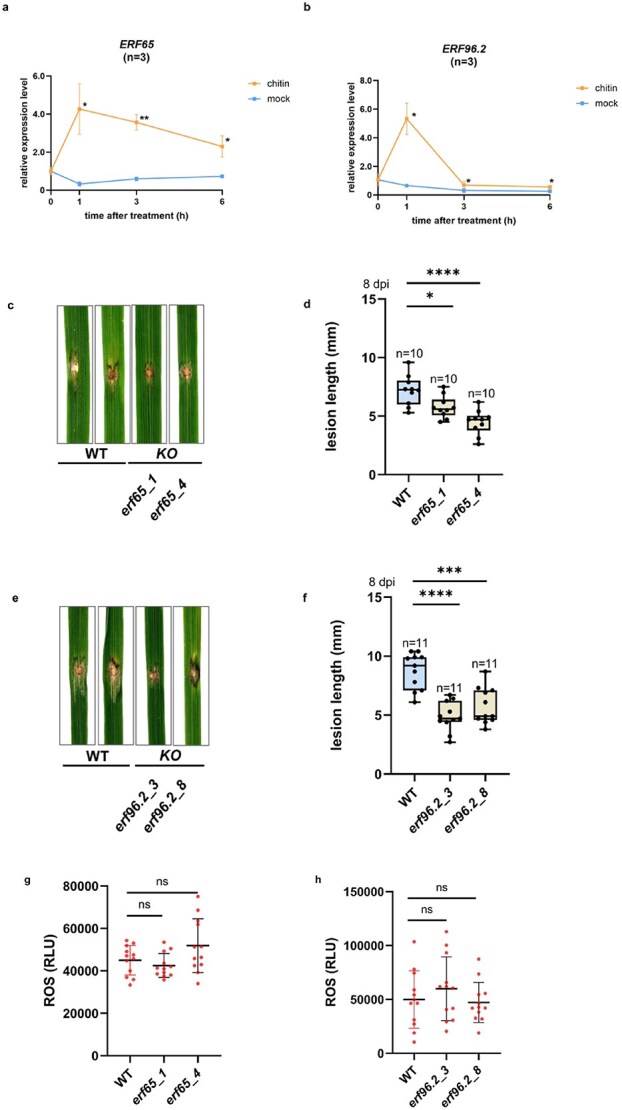
OsERF genes negatively regulate rice immunity against *M. oryzae*. Relative expression of *OsERF65* (a) and *OsERF96.2* (b) induced in suspension cells treated with chitin. Expression levels were quantified by qRT-PCR. Bars represent mean ± SD (*n* = 3 biological replicates; two-tailed *t*-test; ^*^P < 0.05; ^**^P < 0.01). The representative disease symptoms (c) and disease lesion length (d) on the inoculated leaves of the WT and *erf65* transgenic lines after *M. oryzae* punch infection. Bars represent mean ± SD (*n* = 10 biological replicates; unpaired *t*-test; ^*^P < 0.05; ^****^P < 0.0001). The representative disease symptoms (e) and disease lesion length (f) on the inoculated leaves of the WT and *erf96.2* transgenic lines after *M. oryzae* punch infection. Bars represent mean ± SD (*n* = 11 biological replicates; unpaired *t*-test; ^***^P < 0.001; ^****^P < 0.0001). Peak ROS induction of chitin minus mock in WT and *erf65* (g) and *erf96.2* (h) transgenic plants. Leaf discs were treated with 1 μg/mL chitin or mock (ddH_2_O). Bars represent mean ± SD (*n* = 12 biological replicates; unpaired *t*-test). The conidia-induced expression levels of the defense marker genes *OsPR-1a*, *OsPR5*, *OsPR1b*, and *OsPR10* in the WT, *erf65* (i), and *erf96.2* (j) seedlings. Bars represent mean ± SD (*n* = 4 biological replicates; two-way ANOVA, different lowercase letters indicate significant differences, *P* < 0.05). Chitin-induced MAPK phosphorylation in leaf discs of the WT and *erf65* (k) or *erf96.2* (l) lines.

**Figure 9:**
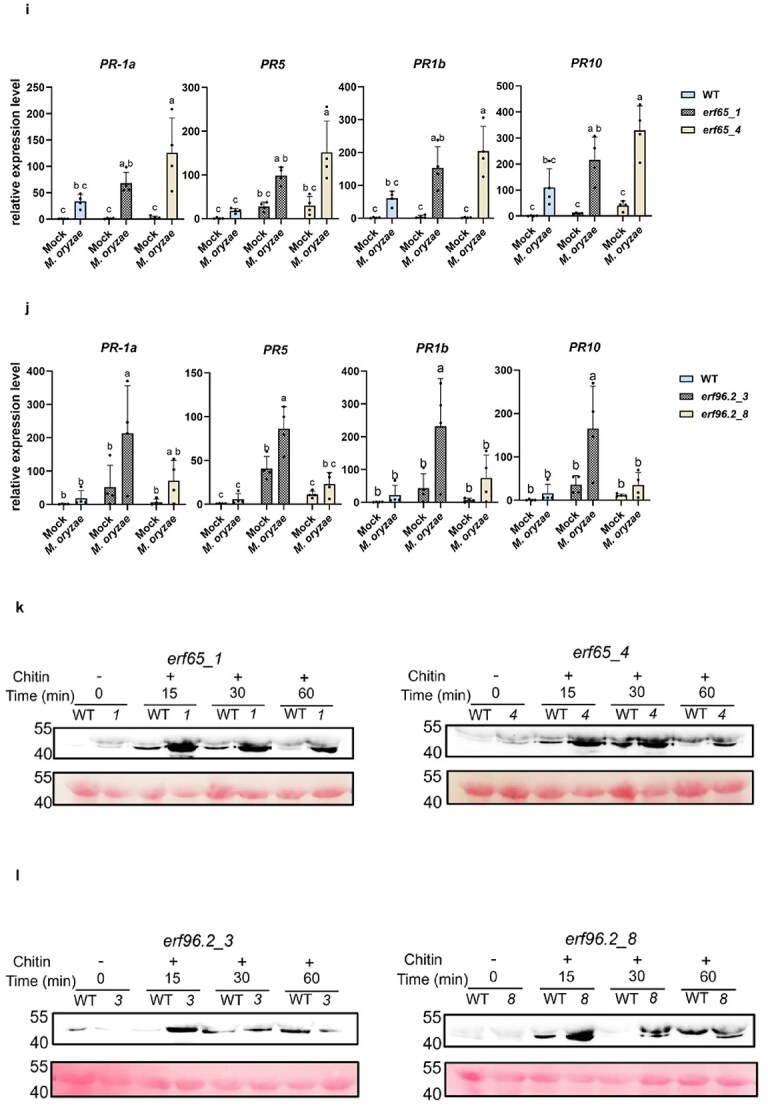
Continued

**Figure 10:**
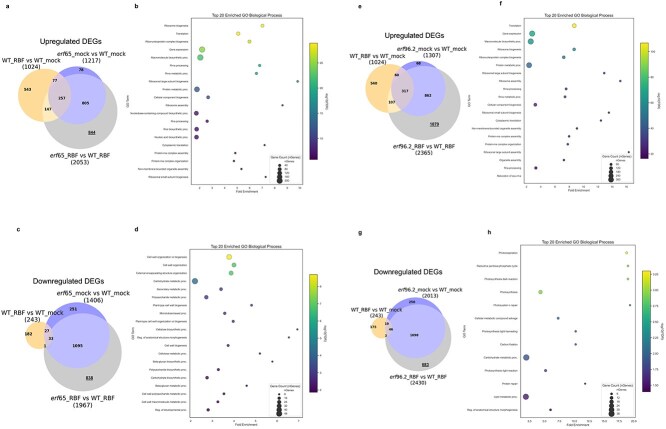
RNA-seq and GO analysis of *erf65* and *erf96.2* in response to *M. oryzae* infection. (a, c, e, g) Identification of *M. oryzae*–responsive DEGs (underlined) in *erf65* (a, c) and *erf96.2* (e, g) relative to WT. (b, d) Top 20 enriched GO Biological Process terms associated with the upregulated (b) and downregulated (d) DEGs in *erf65*. (f, h) Top 20 enriched GO terms for the upregulated (f) and downregulated (h) DEGs in *erf96.2*. RBF, rice blast fungus.

To more deeply decipher the biological pathways regulated by *OsERF65* and *OsERF96.2*, we performed a comparative RNA-seq analysis on the WT plants and the *erf65* and *erf96.2* KO plants following the virulent race (Ao92-06-02) of *M. oryzae* infection (Fig. 10). In the *erf65* mutant, we identified 844 upregulated and 838 downregulated DEGs compared to the WT specifically under *M. oryzae* infection conditions (Fig. 10a and c; underlined). GO enrichment analysis revealed that pathways related to ribosome biogenesis and translation were highly enriched among the upregulated DEGs (Fig. 10b), whereas cell wall organization–related pathways were enriched among the downregulated DEGs (Fig. 10d). Similarly, for *OsERF96.2*, the 1079 upregulated DEGs were enriched in translation and ribosome biogenesis pathways (Fig. 10e and f). However, distinct from *OsERF65*, the 683 downregulated DEGs in *erf96.2* were significantly enriched in photosynthesis-related terms (Fig. 10g and h). Moreover, quantitative reverse transcription PCR (qRT-PCR) experiment during *M. oryzae* infection revealed that the *erf65* and *erf96.2* KO plants exhibited increased expression of multiple defense-related genes (Fig. 9i and j). These results suggest that OsERF65 and OsERF96.2 act as negative regulators of immunity against *M. oryzae*, likely by restricting translation capacity and by repressing the induction of the defense-related genes.

## Discussion

This study addresses a critical knowledge gap by providing a comprehensive transcriptional analysis of key immune regulators, including OsPRR candidates, OsRLCKs, and OsTFs, at the family and subfamily levels, in response to biotic stress. Specific subfamilies have distinct roles in plant immunity; for instance, the RLK-LRR-XII subfamily is primarily involved in MAMP perception, whereas the RLCK-VII subfamily is a key regulator of PTI responses. Building on this, we systematically characterized the differential gene expression of these families in both rice plant and suspension cell experiments. This overlapping DEG system, supplemented with subfamily information, allowed us to identify broadly responding subfamilies and dissect specific transcriptional signatures associated with pathogen- and MAMP-triggered immunity.

Our analysis provides a comprehensive overview of the transcriptional responses of the OsRLK family, which consists of more than 20 subfamilies (Table 1). These results supported the importance of previously identified biotic stress–related subfamilies and highlight several new subfamilies that warrant further investigation. For example, our identification of *OsFLS2* (RLK-LRR-XII) in the overlapping DEG list (Supplementary Table S13 and Fig. 2c) validated our approach, as this gene encodes a well-characterized receptor for bacterial flagellin in rice (Takai et al. 2008). The DLSV subfamily, the largest in the OsRLK family, contained the most DEGs in both rice plant and suspension cell experiments (Fig. 2a and b). DLSV is a complex group containing multiple subgroups (Zheng et al. 2016), and studies have typically focused on specific subgroups within the DLSV (Ngou et al. 2024). The consistent and strong response of DLSV DEGs in plant and suspension cell systems (Fig. 2) underscores their overall importance in immunity and warrants further investigation. A key pattern emerged when we analyzed the 17 RLK subfamilies where at least 30% of the transcripts were DEGs (Table 2). All the previously reported biotic stress–related subfamilies (highlighted in yellow and orange) showed a high proportion of upregulated DEGs (≥27%) and a low proportion of downregulated DEGs (≤6%). Based on this transcriptional signature, LRR-VIII-1, LRR-XI-2, and RKF3 may be promising new candidates with functional roles in MAMP perception and biotic stress. Indeed, our DEG list contained known immune regulators from two of the proposed candidate subfamilies. We identified *OsRIR1* (from LRR-VIII-1) (Fig. 2c) as a negative regulator whose mutant showed increased resistance to *Xoo* (An et al. 2022). We also identified *OsSOBIR1* (from LRR-XI-2) (Fig. 2c), a positive regulator of the PTI response and antiviral defense against rice black-streaked dwarf virus (RBSDV) (Zhang et al. 2021).

In contrast to the OsRLKs, the OsRLP family consisted of fewer subfamilies and showed a clearer DEG distribution, with the LRR subfamily being the most responsive in both experiments (Fig. 3a and b). Our analysis also highlighted the RLP-MALECTIN and RLP-WAK subfamilies because of their high percentages of DEGs (Table 3). Although the function of RLP-MALECTINs in rice is poorly understood, the related RLK-MALECTIN protein, FERONIA, is a well-known regulator of RALF signaling in *Arabidopsis* (Lindner et al. 2012, Haruta et al. 2014, Wang et al. 2022). Similarly, the RLK-WAK protein GhWAK7A acts as a coreceptor for chitin signaling in cotton (Wang et al. 2020b). These associations suggest that the RLP-MALECTIN and RLP-WAK subfamilies identified in our study are strong candidates with novel roles in rice immunity, which require further functional characterization. Notably, well-established chitin receptors, including the RLK *OsCERK1* and the RLPs *OsCEBiP*, *OsLYP4*, and *OsLYP6*, are not present in Figs. 2c and 3c. This is because their expression did not meet the criteria for overlapping DEG selection (Supplementary Table S14); for instance, *OsCERK1* was a DEG in the rice suspension cell experiment only.

We generated phylogenetic trees for both the OsRLK and OsRLP families to visualize the overall distribution of DEGs (Supplementary Figs. S4–S6). Among the LRR subfamilies, LRR-XII (Supplementary Fig. S5; highlighted in red branches) showed a higher proportion of DEGs than the others did. These findings support the well-established role of LRR-XII members in the perception of MAMPs. Among the other RLK and RLP subfamilies, examination of the phylogenetic trees did not reveal any broader patterns or clear associations between the distinct phylogenetic clades and transcriptional responses to immune triggers. This suggests that responsiveness to *M. oryzae* or chitin may be a feature that has evolved in multiple distinct lineages within these large gene families. These findings have several methodological implications. Although phylogenetic trees are invaluable for understanding evolutionary relationships, the sporadic nature of immune gene activation across lineages makes it challenging to identify functionally enriched subfamilies by visual inspection alone. Therefore, we propose that calculating the proportion of DEGs within each subfamily, as performed in this study, offers a more direct and quantitative approach for identifying groups of genes critical to the plant immune response.

Notably, our transcriptomics-based approach did not identify all the RLCK-VII subfamily genes previously linked to chitin immunity, such as *OsRLCK185* and *OsRLCK176* (Supplementary Table S15). A likely explanation is that the functions of these proteins are regulated primarily at the post-transcriptional level (e.g. through phosphorylation) and thus do not exhibit significant changes in transcript abundance under our experimental conditions. This highlighted a known limitation of transcriptomic screening, which is not designed to capture post-transcriptional events, and underscored the importance of integrating multiple omics data types in future studies. Our analysis of OsRLCKs identified the RLCK-IV, -II, and -Os subfamilies as highly responsive to immune triggers, each exhibiting a large proportion of upregulated DEGs (Table 4). Among the identified RLCK subfamily members, only *OsSpl26* (RLCK-Os) has been previously reported to regulate rice immunity and ROS accumulation (Shang et al. 2022). The functions of the remaining members remain uncharacterized. These findings expand upon the well-known role of the RLCK-VII subfamily and highlight these additional groups as promising candidates for future functional analyses of plant immunity.

Regarding OsTFs, our analysis highlighted four key families, WRKY, HSF, NAC, and AP2/ERF, which are strongly implicated in the PTI response to *M. oryzae*. Our combined analysis identified 320 TF DEGs across plant and suspension cell experiments (Fig. 1b). We compared these with a list of WRKY, NAC, and AP2/ERF TFs for which roles in biotic stress have been reported to validate our dataset. This comparison confirmed that TF DEGs identified in the plant and suspension cell experiments (Supplementary Tables S11 and S12) included several known positive regulators (e.g. *OsNAC6* and *OsNAC111*) and negative regulators (e.g. *OsNAC122* and *OsNAC131*) (Nakashima et al. 2007, Sun et al. 2013, Yokotani et al. 2014). Furthermore, our TF DEGs included other key immune-related TFs, including the hypersensitive cell death regulator *OsNAC4* (Kaneda et al. 2009) and the defense-inducible gene *OsBIERF1* (Cao et al. 2006). Among these OsTFs, *OsNAC111* and *OsBIERF1* were included in the overlapping DEGs (Supplementary Table S13). The successful identification of these known regulators, coupled with the functional validation of our novel candidates, indicates that our selection method was sufficiently stringent to reliably prioritize genes with functionally relevant roles in immunity. Notably, the inclusion of the HSF family was unexpected, given its well-characterized role in abiotic stress responses (Tao et al. 2024). Although the HSF family had the highest percentage of DEGs (34%) (Table 5), its transcriptional signature was distinct from that of the other TF families (Fig. 5c). It displayed a balanced proportion of both upregulated (26%) and downregulated (26%) DEGs, in contrast to the predominantly upregulated patterns observed in WRKY, NAC, and AP2/ERF (Table 5). This distinct regulatory pattern may reflect the complex crosstalk between biotic and abiotic stress signaling, suggesting that plants must carefully modulate HSF activity to balance their defense against environmental stress responses.

In summary, this study provided a multilayered view of rice immune response at the family and subfamily levels. Our transcriptional analysis confirmed the involvement of previously characterized OsPRR, OsRLCK, and OsTF subfamilies and systematically identified a wealth of novel, highly responsive subfamilies and candidate genes. These findings offer valuable resources and lay the groundwork for future functional studies aimed at dissecting the complex regulatory networks governing plant immunity.

In addition to providing the transcriptional landscape, this study presents a robust pipeline for gene selection and functional validation. Although bioinformatics analysis of RNA-seq data is a common approach, selecting candidates is often challenging because of the large number of DEGs and ambiguous GO results. Our work demonstrates that the use of overlapping DEGs from the rice plant and suspension cell experiments is a reliable system for prioritizing critical genes. The efficacy of this strategy was confirmed by the functional validation of four candidates, which showed that two RLCKs, OsRLCK298 and OsBSR1, act as positive regulators. In contrast, two ERF TFs, OsERF65 and OsERF96.2, act as negative regulators of immunity against *M. oryzae*. Interestingly, GO terms associated with translation and ribosome biogenesis were enriched in both the *erf65* and *erf96.2* KO mutants (Fig. 10b and f). De-repression of pre-existing mRNA translation is a key mechanism enabling rapid immune responses following pathogen challenge. For example, translation of TBF1, a key immune transcription factor governing the growth-to-defense transition, is regulated by two upstream open reading frames (uORFs) upon immune induction (Pajerowska-Mukhtar et al. 2012). Consistently, the suppressive effects of uORFs and R-motifs on translation of PTI-associated genes are alleviated upon treatment with the elicitor peptide elf18 (Xu et al. 2017). It is possible that OsERF65 and OsERF96.2 suppress rice immunity by switching off pre-existing mRNA translation, and that the *erf65* and *erf96.2* KO mutants achieve enhanced resistance to *M. oryzae* by increasing the overall translational capacity. OsERF65 appears to support cell wall–related processes, while OsERF96.2 facilitates photosynthesis (Fig. 10d and h). Since translation is an energy-intensive process, it may normally be suppressed by these two ERFs to prioritize plant growth by allocating resources to essential physiological processes, including cell wall biogenesis (Takagi et al. 2022) and photosynthesis (Huot et al. 2014). Thus, OsERF65 and OsERF96.2 may function as specific “brakes” that prevent excessive investment in ribosome biogenesis and translation during immune activation, thereby avoiding growth penalties. Regarding the growth phenotypes and breeding relevance, *OsERF65* KO lines were reported to have compromised plant height but enhanced yield (Xie et al. 2023). In contrast, no observable phenotypic differences were reported for *OsERF96.2* mutant lines compared to the WT (Sun et al. 2022a). Therefore, *OsERF65* and *OsERF96.2* represent suitable targets for gene editing to improve disease resistance without incurring significant agronomic penalties. Notably, three of these genes (*OsRLCK298*, *OsERF65*, and *OsERF96.2*) have no previously reported roles in rice blast resistance.

Identification of OsRLCK298 as a positive regulator of immunity is particularly noteworthy. OsRLCK298 belongs to the RLCK-VII-6 subfamily, which has been implicated in the negative regulation of flg22-induced ROS signaling (Rao et al. 2018). Our finding that members of this subfamily positively regulated immunity against fungal pathogens suggests that these proteins may have divergent or context-dependent roles. Therefore, OsRLCK298 may also function in flg22-triggered immunity against bacterial pathogens, a hypothesis that warrants further investigation.

The AP2/ERF transcription factor family is more frequently associated with abiotic stress than with biotic stress (Xie et al. 2022). Although rice contains 188 genes (Table 1), only a few, such as *OsRap2.6* (positive regulator) and *OsERF922* (negative regulator), are associated with rice blast disease (Wamaitha et al. 2012, Liu et al. 2012b). The roles of most family members in this context remain unknown. Our study contributes to this understanding by demonstrating that OsERF65 and OsERF96.2 also act as negative regulators of rice immunity. This finding is consistent with a recent report on *OsERF65*’s role in sheath blight resistance (Xie et al. 2023), suggesting a broader function for this gene in regulating the defense against multiple fungal pathogens.

The pipeline developed in our study, which uses overlapping DEGs from plant and suspension cell experiments, may be a valuable tool for identifying key immune regulators of other pathogens. The case of *OsBSR1* exemplifies this potential; this candidate gene is known to be involved in resistance not only to another fungal disease (*Cochliobolus miyabeanus*), but also to bacterial (*Burkholderia glumae*) and viral (rice stripe virus) diseases (Maeda et al. 2016). The successful identification of this broad-spectrum regulator supports the reliability of our selection method. The comprehensive list of overlapping DEGs (Supplementary Table S13) offers a valuable resource for the community. In addition to the primary gene families studied, this dataset also contained other essential immune components such as *OsPep3*. This gene encodes a damage-associated molecular pattern (DAMP), a type of “danger signal” released from the plant’s own cells upon injury, as opposed to the MAMPs that originate from microbes. The presence of this key DAMP, which confers resistance to multiple biotic threats (Shen et al. 2022), further underscores the importance of our dataset.

We also identified potential PRR complex candidates. We hypothesized that new chitin-perceiving PRRs would form complexes with members of our overlapping RLK and RLP candidate pools. Given the large number of possible combinations, we first employed an *in silico* approach using LocalColabFold to predict protein–protein interactions (PPI) and prioritize candidates. We successfully predicted experimentally confirmed interactions between OsCERK1 and its coreceptors, OsLYP4 and OsLYP6, which served as positive controls (PPI score > 0.75; Supplementary Table S16) to validate this method. Following this validation, we predicted the interactions between the 63 overlapping RLK candidates and 6 overlapping RLP candidates.

A significant proportion (67%) of the 573 combinations tested yielded positive interaction scores, indicating a high potential for complex formation among these candidates (Supplementary Table S17). These predicted interactions were visualized in a protein interaction network, where RLKs (red) and RLPs (blue) were represented as nodes (Supplementary Website 1). The large number of positive predictions highlighted the need for additional filtering criteria to further refine the list of promising OsPRR candidates. Ultimately, experimental validation, such as coimmunoprecipitation or split-luciferase assays, is required to confirm these *in silico* predictions and decipher the functional roles of these high-confidence receptor complexes in MAMP perception and PTI responses.

In conclusion, we provide a systematic view of the transcriptional landscape of key PTI regulators in rice. We successfully identified subfamilies with enriched expression of defense-responsive genes, and the functional analysis confirmed that the candidates identified in our pipeline were genuine regulators of immunity against *M. oryzae*. The comprehensive DEG datasets and validated selection strategy established in this study provide a valuable resource that will facilitate future research on the complex regulatory networks of biotic and abiotic stress responses in critical crops.

## Materials and Methods

### Transcripts for PRR candidates, RLCKs, and TFs

Transcript IDs of OsRLKs, OsRLCKs, and OsTFs were obtained from the iTAK website (https://itak.feilab.net/cgi-bin/itak/db_browse.cgi, accessed 24 April 2024). Additionally, OsRLKs and OsRLPs were also identified through the pipeline (Restrepo-Montoya et al. 2020) using the downloadable protein file all.pep (https://rice.uga.edu/pub/data/Eukaryotic_Projects/o_sativa/annotation_dbs/pseudomolecules/version_7.0/all.dir/, accessed 28 April 2022) as the input database. All the transcripts were carefully checked within each category, and duplicates were removed. Finally, GPI-anchored RLP proteins were added by filtering all.pep using “GPI-anchored protein.”

### RNA-seq analysis of *M. oryzae* infection and chitin treatment

RNA isolation and RNA-seq library preparation were performed as described previously (Wang et al. 2020a). The RNA-seq dataset is available in the Gene Expression Omnibus (GEO) under accession number GSE252769. For this dataset, we conducted DEG analysis on raw fastq files. Quality checks were done using FastQC (v.0.11.9), and contaminations, such as ribosomal RNAs, were removed using BBduk (v.38.87). Illumina adapters were removed using cutadapt (v.2.8). The reference rice genome of Os-Nipponbare-Reference-IRGSP-1.0 (https://rapdb.dna.affrc.go.jp/download/irgsp1.html) was used to generate a decoy-aware transcriptome using Salmon. We used Osativa_323_v7.0.gene_exons.gff3 (https://phytozome-next.jgi.doe.gov/) as the annotation file. The estimated counts were imported through tximportData (v.1.16.0). The R package (v.4.0.2) DESeq2 (v.1.28.1) was used to identify DEGs. The DEG threshold was set at a false discovery rate (FDR/*P*_adj_) < 0.05 and an absolute log2FoldChange > 1. In this experiment, we used the transcript-level quantification for DEG analysis since it offers a higher resolution at the isoform level. All the tools were used with default settings unless otherwise specified.

### RNA-seq analysis of *erf* mutants

The RNA-seq dataset is available in the GEO under accession number GSE316765. We employed a comprehensive transcript-merged pipeline. This combined reference strategy was adopted after preliminary comparative analysis demonstrated that integrating transcript models from both databases yielded improved expression profiles and superior sample clustering in PCA compared to using the Rice Annotation Project Database (RAP-DB) alone. Read quality control and trimming were performed using fastp (v.0.23.4) with the flags --cut_front --cut_tail --cut_right --correction (Chen et al. 2018). Clean reads were mapped using a hybrid alignment strategy with STAR (v.2.7.11b) (Dobin et al. 2013) and Bowtie2 (v.2.5.4) (Langmead and Salzberg 2012). The Nipponbare reference genome (IRGSP-1.0) and its associated reference transcripts were utilized for all read mapping (Kawahara et al. 2013, Sakai et al. 2013). To ensure a comprehensive reference, the transcript model set was primarily obtained from the RAP-DB, supplemented with nonredundant transcript models from the Rice Genome Annotation Project (RGAP/MSU) database. The reference genome and merged transcript dataset were indexed by STAR with the settings --runMode genomeGenerate, --genomeSAindexNbases 13, and --sjdbOverhang 149, supplementing the transcript database via the --sjdbGTFfile argument. Bowtie2 indices were generated using default parameters. Read mapping by STAR was performed with the following parameters: --outSAMtype BAM SortedByCoordinate, --quantMode TranscriptomeSAM, --outSAMunmapped Within KeepPairs, and --outFilterMultimapNmax 1000. Unmapped reads were extracted from the STAR-generated BAM files using Samtools (v.1.19.2) with the settings -h -f 13 (Danecek et al. 2021) and subsequently aligned to reference transcripts using Bowtie2 under default settings. The resulting alignment files were merged into a single BAM file per sample using samtools merge. Transcript abundance was estimated using Salmon (Patro et al. 2017) with default parameters, providing the transcript database via the -t argument. Quantification data were imported into the R environment (R Core Team 2026) using tximport with default settings (Soneson et al. 2016). DEG analysis was conducted using the DESeq2 package (Love et al. 2014). A regression model was applied to evaluate the effect of treatment (mock vs *M. oryzae* infection) separately at each time point. Independent hypothesis weighting (IHW) (Ignatiadis et al. 2016) was employed for *P*-value adjustment. DEGs were identified using thresholds of |log2FC| ≥ 1 and an adjusted *P*-value (*P*_adj_) < 0.05.

### Plant materials and suspension cells

Rice plants (*Oryza sativa* L. ssp. *japonica* cv. Nipponbare) were grown on shelves under long-day conditions (16:8 h, light:dark, 25°C). Two knockout lines each of *erf65* and *erf96.2* were generated using the CRISPR/Cas9 system (Mikami et al. 2015). Two overexpressing lines of *OsRLCK298_*OX were generated by introducing coding sequences of *OsRLCK298* into the pENTR/D-Topo vector, followed by p2k Gateway cloning. *Agrobacterium*-mediated transformation of rice calli was performed according to an established method (Hiei et al. 1994).

Experimental setups and treatments for the rice plant experiment and the suspension cell experiment were previously described (Wang et al. 2020a). For candidate gene induction, rice suspension cells derived from Nipponbare calli were grown in R2 medium (Hayakawa et al. 1992). Rice suspension cells were cultured in R2 medium and covered for overnight incubation and then treated with 2 μg/mL chitin (hexaacetylchitohexaose, Biosynth, OH07433) or phosphate-buffered saline as a mock.

### Punch infection assay

Four-week-old rice plants’ fourth leaves were punch inoculated with an *M. oryzae* (virulent race Ao92-06-02) conidia suspension of 10^5^ conidia/mL in 0.01% Tween-20 (Shimizu et al. 2022). Disease lesions were scanned at 8 or 9 dpi. Three independent experiments were performed in which, each time, at least 10 plants were used for punch inoculation.

### Spray infection assay

Twenty-eight-day-old rice seedlings were sprayed with an *M. oryzae* (virulent race Ao92-06-02) conidia suspension of 2 × 10^5^ conidia/mL or a mock treatment (ddH_2_O). Plants were placed into a dark dew chamber. At 24 hpi, plants were removed from the chamber and returned to half lighting intensity (16:8, light:dark). The third leaves were harvested at 48 hpi; one biological sample was a pool of five leaves from five individual seedlings. Two independent experiments were performed in which 20 leaves (4 biological samples) per genotype were used for each treatment.

### Measurement of ROS levels

ROS levels were determined based on chemiluminescence measured by a multimode reader (TriStar2 LB 942). Chitin treatment and ROS measurement were conducted following established protocols (Li et al. 2024) with minor adjustments. Leaf discs were placed on a 96-well plate. Three leaf discs per well from 5-week-old plants’ fifth leaves and three individual plant leaves per genotype were used. In total, 12 wells per genotype (WT and mutant lines) were used per treatment (chitin and mock). Buffer [ddH_2_O: 20 mM L-012 (Fujifilm Wako, 120-04891): 1 mg/mL peroxidase (TOYOBO, PEO-131), 96.5:2.5:1] was used to replace ddH_2_O water after 24 h incubation, leaf discs were covered in dark and allowed to rest for 30 min, and background chemiluminescence was measured for 10 min before 1 μg/mL chitin (hexaacetylchitohexaose, Biosynth, OH07433) and mock (ddH_2_O) were added. Luminescence was measured for a total of 90 min. Peak level of chitin minus mock was used.

### MAPK phosphorylation assay

Leaf discs (3 mm diameter) were excised from the fourth or fifth leaves of rice plants. Leaf discs were floated in incubation buffer (ddH_2_O containing 0.05% Silwet L-77) overnight to allow recovery from wounding stress. The buffer was replaced with fresh incubation buffer, followed by a 30 min equilibration period. Samples were harvested at 0, 15, 30, and 60 min after treatment with 10 μg/mL chitin (hexaacetylchitohexaose; Biosynth, #OH07433). For the 0 min control, samples were harvested immediately prior to treatment. All the samples were flash-frozen in liquid nitrogen, ground to a fine powder, and homogenized in 1× Laemmli extraction buffer (62.5 mM Tris–HCl pH 6.8, 1% SDS, 10% glycerol, 5% β-mercaptoethanol, and 0.003% bromophenol blue). Phosphorylated MAPK proteins were detected by immunoblotting using an anti-Phospho-p44/42 MAPK (Erk1/2) antibody (Cell Signaling Technology, #4370). Two independent experiments were performed; for each time point, samples consisted of a pool of 24 leaf discs derived from three individual plants (8 discs per plant).

### RNA isolation and qRT–PCR

Total RNA was isolated by the Trizol method, and cDNA was synthesized using the ReverTra Ace qPCR RT Master Mix with gDNA Remover kit (TOYOBO, FSQ-301). For qRT-PCR, gene-specific primers were designed (Supplementary Table S18). *OsUbq* was used as an internal control to normalize gene expression. Reactions were performed using FastStart Essential DNA Green Master kit (Roche). Relative expression level values were calculated using the 2^−ΔΔ*CT*^ method (Livak and Schmittgen 2001).

### Phylogenetic analysis

The amino acid sequences of OsRLKs and OsRLPs were obtained from all.pep. All the protein sequences were aligned using Clustal Omega (https://www.ebi.ac.uk/jdispatcher/msa/clustalo). Phylogenetic analysis was performed with iqtree2 (http://www.iqtree.org/) using the maximum likelihood method with 1000 bootstrap replications. iTOL (https://itol.embl.de/) was used to draw the phylogenetic tree. The phylogenetic tree was midpoint-rooted. The circular tree was used for better visualization of DEG distribution.

## Supplementary Material

Supplementary_Figs_S1-S6_pcag019

Supplementary_Tables_S1-S18_pcag019

Supplementary_website_1_rlk_rlp_interaction_result_WW_pcag019

## Data Availability

We deposited RNA seq data and our pipeline of bioinformatic analysis in the GEO (GSE252769 and GSE316765). All the data supporting the findings of this study are available in the main text or in the Supplementary Data. Source data are provided with this article. Sequence data from this article can be found in the rice genome databases under the following locus identifiers: *OsBIERF1* (LOC_Os09g26420.5), *OsBSR1* (LOC_Os09g36320.1), *OsCEBiP* (LOC_Os03g04110.1), *OsCERK1* (Os08g0538300–01), *OsERF65* (LOC_Os07g42510.1), *OsERF96.2* (LOC_Os10g41330.2), *OsFLS2* (LOC_Os04g52780.1), *OsLYP4* (LOC_Os09g27890.1), *OsLYP6* (LOC_Os06g10660.1), *OsNAC4* (LOC_Os01g60020.1), *OsNAC6* (LOC_Os01g66120.3), *OsNAC111* (LOC_Os11g05614.1), *ONAC122* (LOC_Os11g03300.1), *ONAC131* (LOC_Os12g03040.1), *OsPep3* (LOC_Os08g07660.1), *PR1b* (Os01g0382000), *OsPR5* (Os12g0628600), *PR10* (Os12g0555000), *OsPR-1a* (LOC_Os07g03710.1), *OsRIR1* (LOC_Os12g10740.1), *OsRLCK298* (LOC_Os10g25550.1), *OsSOBIR1* (LOC_Os06g18000.1), *OsSpl26* (LOC_Os07g04820.1), and *OsUbq* (LOC_Os03g13170.1).
