# Supplementary material for: Subfamily-level comparative transcriptomics of key immune regulators in plants and suspension cells reveals novel rice blast resistance genes
Source: Plant Cell Physiol. 2026 Feb 10;67(6):976–1001. doi: 10.1093/pcp/pcag019 (PMC13317940; doi:10.1093/pcp/pcag019)
Supplement: Supplementary_Figs_S1-S6_pcag019 [file supplementary_figs_s1-s6_pcag019.pptx]

## Slide 1
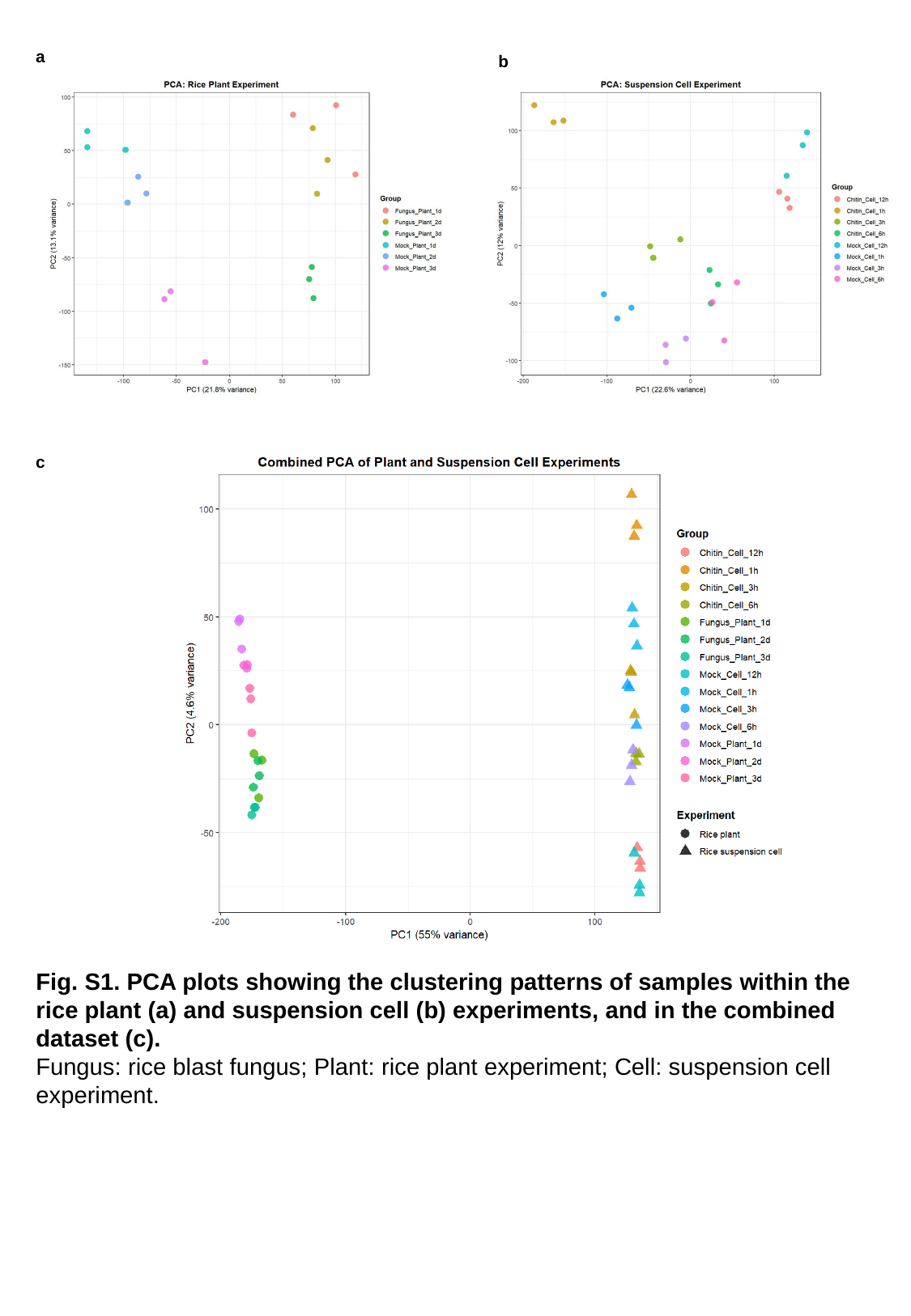

a
b
c
Fig. S1. PCA plots showing the clustering patterns of samples within the rice plant (a) and suspension cell (b) experiments, and in the combined dataset (c).
Fungus: rice blast fungus; Plant: rice plant experiment; Cell: suspension cell experiment.

## Slide 2
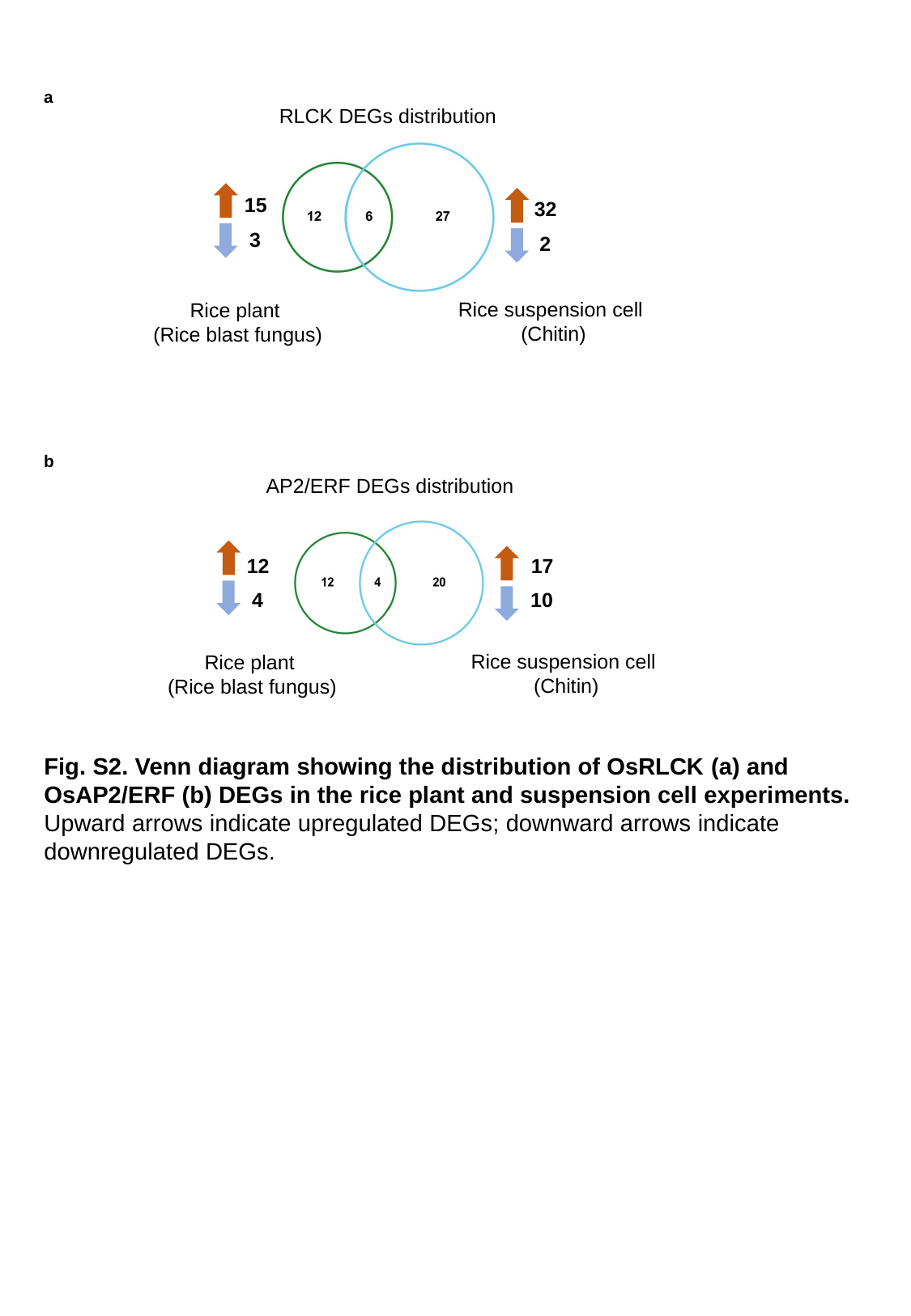

a
RLCK DEGs distribution
15
3
32
2
Rice suspension cell
(Chitin)
Rice plant
(Rice blast fungus)
b
AP2/ERF DEGs distribution
12
4
17
10
Rice suspension cell
(Chitin)
Rice plant
(Rice blast fungus)
Fig. S2. Venn diagram showing the distribution of OsRLCK (a) and OsAP2/ERF (b) DEGs in the rice plant and suspension cell experiments.
Upward arrows indicate upregulated DEGs; downward arrows indicate downregulated DEGs.

## Slide 3
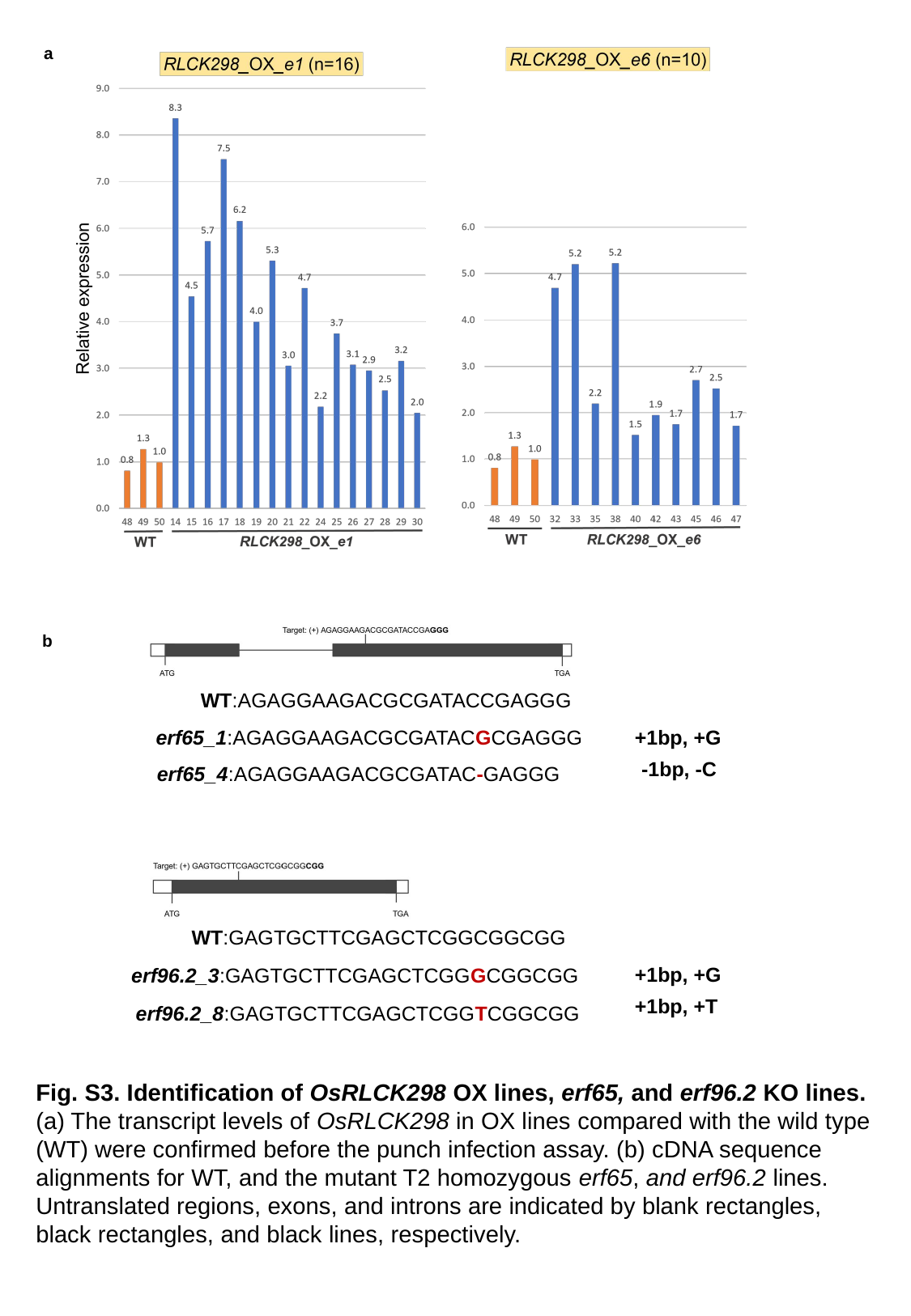

a
WT:AGAGGAAGACGCGATACCGAGGG
erf65_1:AGAGGAAGACGCGATACGCGAGGG
erf65_4:AGAGGAAGACGCGATAC-GAGGG
+1bp, +G
-1bp, -C
b
WT:GAGTGCTTCGAGCTCGGCGGCGG
erf96.2_3:GAGTGCTTCGAGCTCGGGCGGCGG
erf96.2_8:GAGTGCTTCGAGCTCGGTCGGCGG
+1bp, +G
+1bp, +T
Fig. S3. Identification of OsRLCK298 OX lines, erf65, and erf96.2 KO lines. (a) The transcript levels of OsRLCK298 in OX lines compared with the wild type (WT) were confirmed before the punch infection assay. (b) cDNA sequence alignments for WT, and the mutant T2 homozygous erf65, and erf96.2 lines. Untranslated regions, exons, and introns are indicated by blank rectangles, black rectangles, and black lines, respectively.

## Slide 4
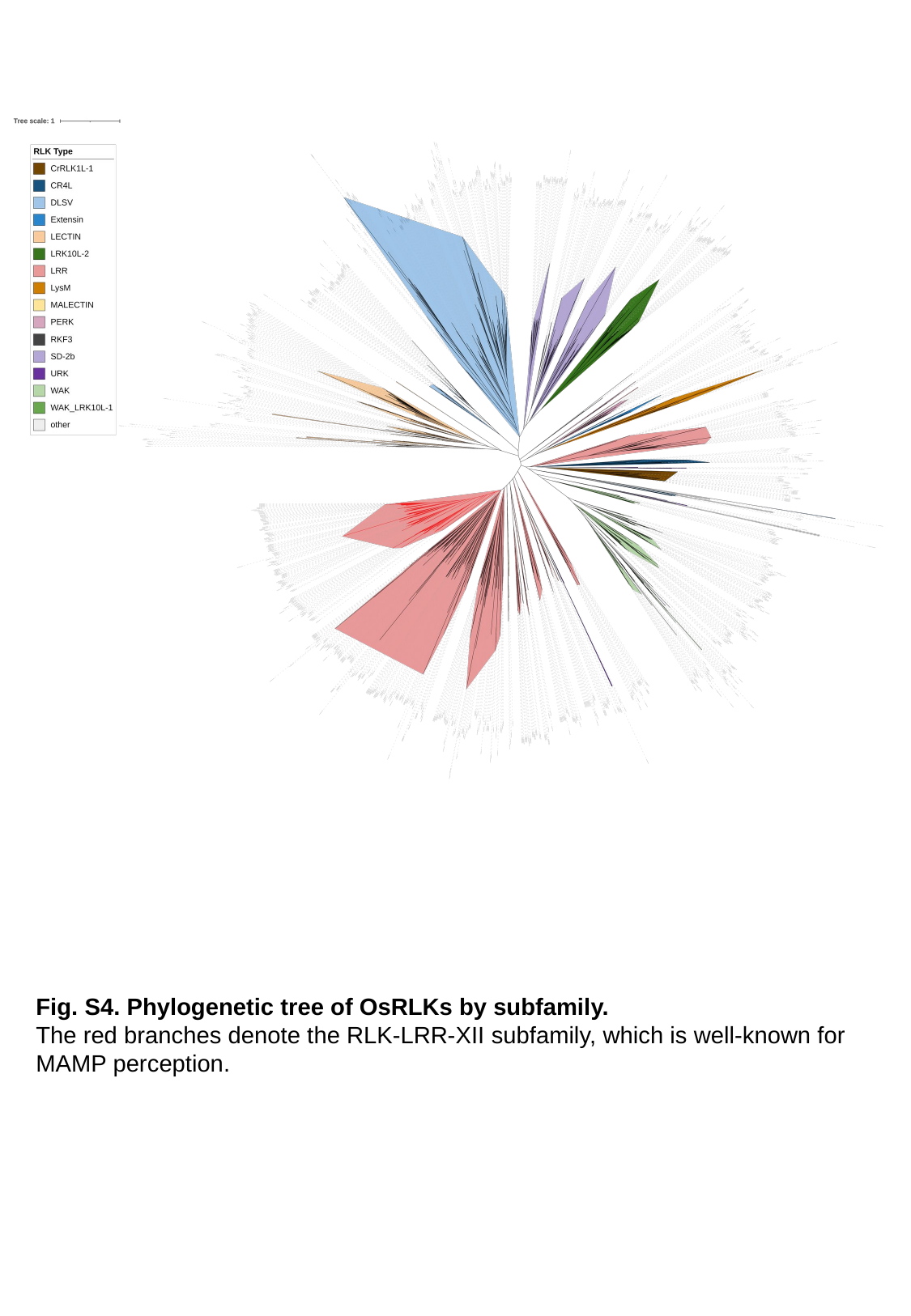

Fig. S4. Phylogenetic tree of OsRLKs by subfamily.
The red branches denote the RLK-LRR-XII subfamily, which is well-known for MAMP perception.

## Slide 5
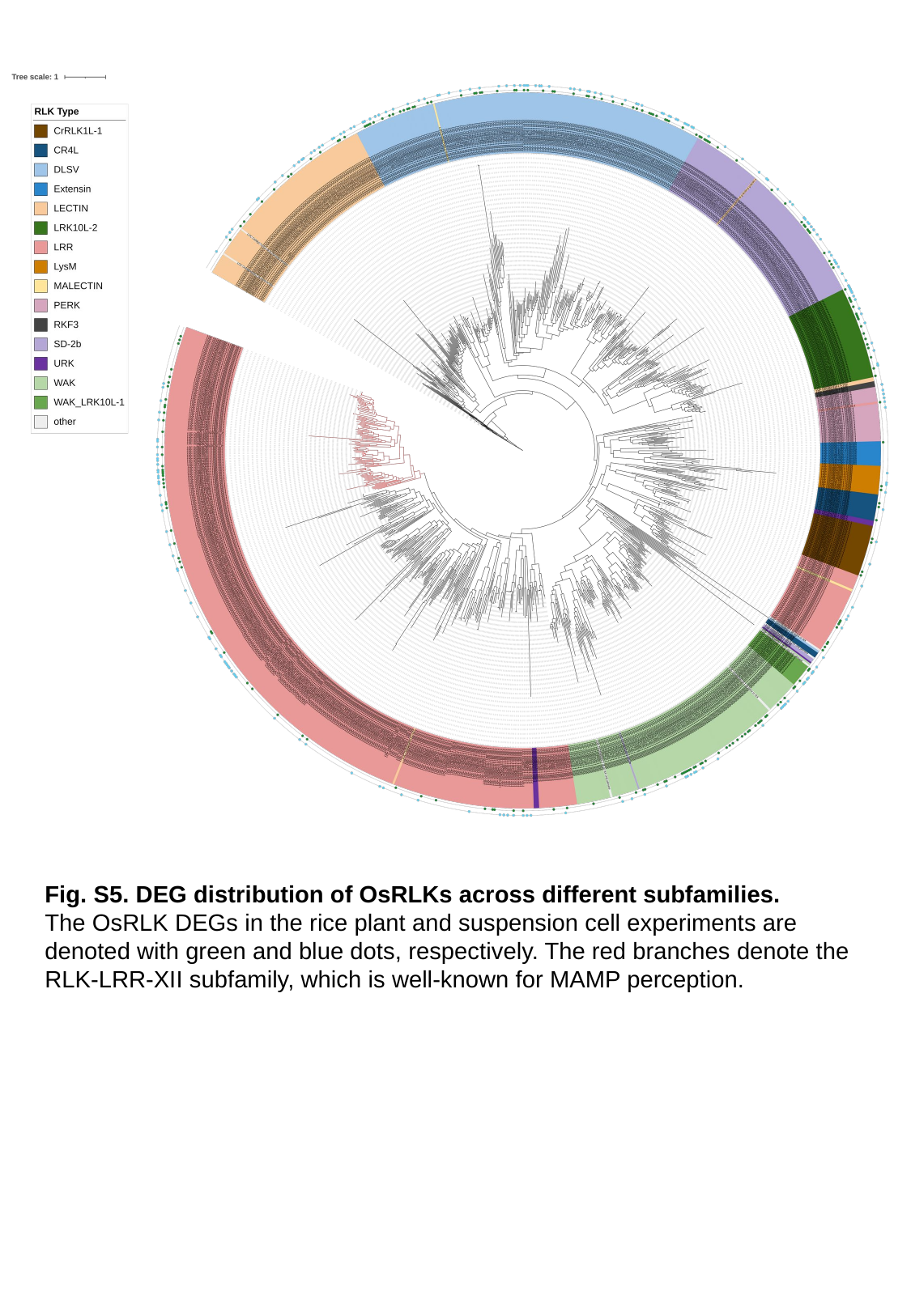

Fig. S5. DEG distribution of OsRLKs across different subfamilies.
The OsRLK DEGs in the rice plant and suspension cell experiments are denoted with green and blue dots, respectively. The red branches denote the RLK-LRR-XII subfamily, which is well-known for MAMP perception.

## Slide 6
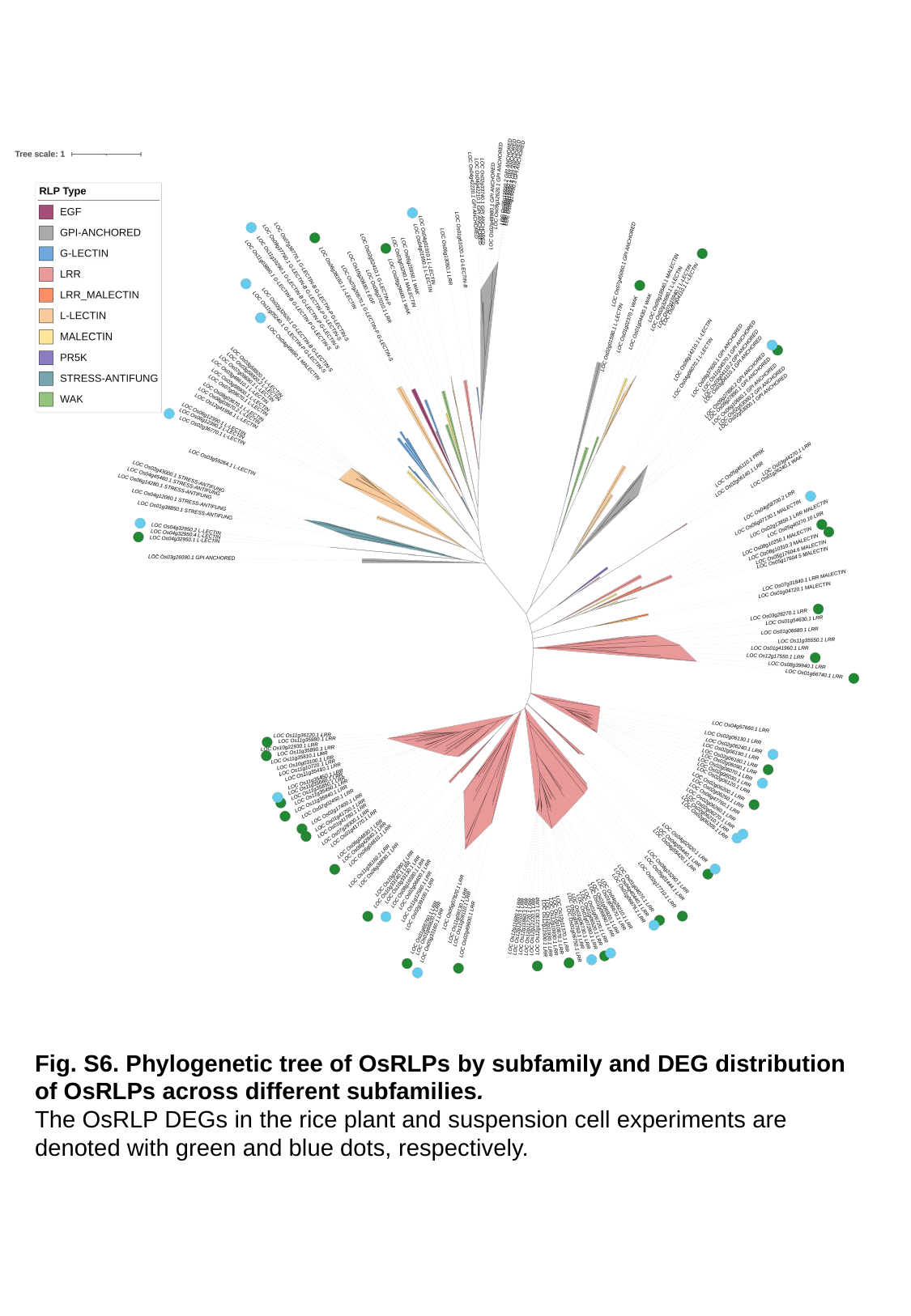

Fig. S6. Phylogenetic tree of OsRLPs by subfamily and DEG distribution of OsRLPs across different subfamilies.
The OsRLP DEGs in the rice plant and suspension cell experiments are denoted with green and blue dots, respectively.
